# Selective Separation of C_8_ Aromatics by an Interpenetrating Metal–Organic Framework Material

**DOI:** 10.1021/acs.inorgchem.4c02969

**Published:** 2024-09-27

**Authors:** Na Sun, Xue Zhou, Han Yu, Xiuwen Si, Fu Ding, Yaguang Sun, Michael J. Zaworotko

**Affiliations:** † Key Laboratory of Inorganic Molecule-Based Chemistry of Liaoning Province, Shenyang University of Chemical Technology, Shenyang 110142, China; ‡ School of Materials Science and Engineering National Institute for Advanced Materials TKL of Metal and Molecule-Based Material Chemistry, Nankai University, Tianjin 300350, China; § Department of Chemical Sciences and Bernal Institute, University of Limerick, Limerick V94 T9PX, Republic of Ireland; ∥ Petrochemical Department, Liaoning Petrochemical College, Jinzhou 121001, China

## Abstract

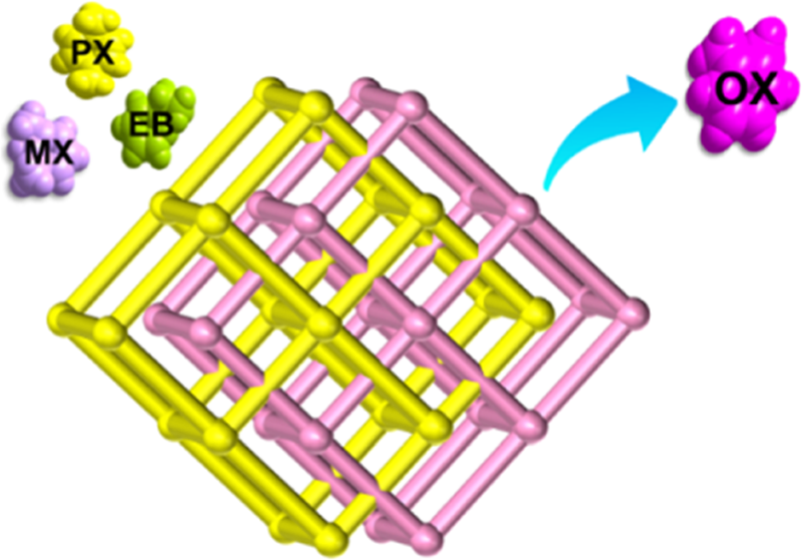

O-xylene (OX) is an important chemical raw material, but it is often produced in mixtures with other C_8_ aromatics. Similar physicochemical properties of the C_8_ isomers make their separation and purification very difficult and energy intensive. There is an unmet need for an adsorbent that would be effective for the separation of OX from the other C_8_ isomers. This work reports a three-dimensional interpenetrated metal–organic framework, SYUCT-110, that interacts with each of the single-component C_8_ isomers to form. The selectivity of C_8_ aromatic hydrocarbons was determined through liquid-phase batch uptake experiments. The results revealed that the selectivity order was OX > PX > MX > ethylbenzene (EB). The selectivity values were found to be 2.63, 1.58, 5.51, 3.71, 1.86, and 3.02 for OX/MX, OX/PX, OX/EB, PX/MX, MX/EB, and PX/EB, respectively. The adsorption capacity of OX was 71 mg/g. Grand Canonical Monte Carlo simulations were used to study the C_8_ adsorption sites, revealing that π···π interactions are the main reason for the observed adsorption selectivity. The adsorption energy calculation results also verified the selectivity of SYUCT-110 for the synthesis of OX.

## Abstract 2

A three-dimensional interpenetrating metal–organic framework material (SYUCT-110) comprising one-dimensional diamond-shaped channels. Due to the weak interaction between SYUCT-110 and C_8_ isomers, it exhibits selective separation of C_8_ aromatics. SYUCT-110 can selectively separate o-xylene from C_8_ aromatics. It achieves a notable selectivity of 5.5 for the OX/EB liquid-phase mixture.

## 1 Introduction

The C_8_ alkyl-aromatic compounds *o*-xylene (OX), *m*-xylene (MX), *p*-xylene (PX), and ethylbenzene (EB) are industrial commodities each of which can be further processed into higher value chemical products.^1,2^ For example, OX is used to produce phthalic anhydride, an important monomer in the production of plasticizers for polyvinyl chloride.^3^ OX is difficult to separate, purify, and expensive to produce.^4^ Indeed, C_8_ separation is recognized as one of the most challenging industrial separations because of the similar physicochemical properties of the C_8_ isomers.^5^ In particular, the similar boiling points of their boiling points make distillation infeasible (OX, MX, PX, and EB are 144.4, 139.1, 138.4, and 136.2 °C, respectively).^6^ Separation methods for C_8_ isomers include crystallization and adsorptive separation.^7^ The crystallization approach uses the difference in melting points for separation, but with a recovery rate and large energy footprint.^8^ Adsorptive separation is the main method of C_8_ isomer separation because of its relatively low energy consumption.^9^ Zeolites, covalent organic frameworks, and porous organic cages have been studied in this context.^10,11^ Unfortunately, such adsorbents tend to have disadvantages such as inflexible structures and low selectivity.^12−14^ Therefore, an adsorption material with controllable structure and high selectivity is needed to address C_8_ isomer separations.

Metal–organic frameworks (MOFs) are coordination networks typically composed of metal cations as nodes and organic ligands as linkers or connectors.^15−18^ MOFs offer large uptake capacity, high specific surface area, and controllable structure, and they have been studied for the separation of C_8_ aromatics.^19−21^ Lower dimensionality coordination polymers have also been studied in this context, e.g., Li et al. prepared a one-dimensional (1D) coordination polymer Mn-dhbq, which can expand with temperature and enable, separation of adjacent meta, and para xylene gas and liquid phases.^22^ The layered material **sql-1-Co-NCS** reported by Wang et al. can undergo switching to a phase loaded with C_8_ aromatics that offers benchmark OX selectivity and high adsorption capacity.^23^**sql-4,5-Zn** reported by Gao et al. has high adsorption capacity for liquid-phase C_8_ aromatics and is the first adsorbent to exhibit higher selectivity for PX, MX, and EB vs OX in binary, ternary, and quaternary mixtures.^24^ The NIIC-30 (Ph) report prepared by Sapianik et al. has a curved channel decorated with aromatic adsorption sites and was found to exhibit a new benchmark for OX/MX separation.^25^ Zhao et al. prepared aluminum-based MOFs that preferentially sorbed OX.^26^ Zhou et al. synthesized ZUL-C3 with a nonaromatic closed pore environment by constructing a mixed polycyclic alkane-type ligand.^27^ Simultaneous separation of four isomers was successfully achieved, and the basis for the dynamic separation of OX/PX and OX/MX was laid.

Although many MOFs have been used to separate C_8_ aromatics, interpenetrating structures have not been studied in this context. MOFs are rich in voids, but their pore structure is not necessarily stable. “Interpenetration” is the phenomenon whereby pores are filled by an adjacent network and the network that is then entangled.^28^ Interpenetration in MOFs is often seen as a negative phenomenon because it necessarily means a reduction in porosity.^29^ However, interpenetration can result in a mechanism to adjust pore size and/or improve the stability of MOFs.^30^ Interpenetration can improve the selectivity and separation ability of materials to a certain extent.^31,32^ Heo et al. prepared a Cu-based metal–organic framework (MOF-14) with doubly interpenetrating structure and studied its adsorption and separation properties for ethane and ethylene.^33^ The results show that the interpenetration can reduce the pore size and make the material have higher ethane selectivity. The novel interpenetrating Zn-MOF (UPC-98) prepared by Wang et al. has good stability and can remain stable in an air environment at 250 °C.^34^ In addition, C_2_H_4_ can be separated from C_2_H_2_ with a purity of up to 99.9%.

We report herein the three-dimensional (3D) interpenetrating MOF SYUCT-110 (SYUCT = Shenyang University of Chemical Technology). X-ray crystallographic analysis revealed a structure with 1D rhomboid channels with moderate dimensions. Adsorptive selectivity was determined by ^1^H nuclear magnetic resonance (^1^H NMR) in batch adsorption experiments. The results showed that SYUCT-110 is selective to OX with the following overall order of selectivity: OX > PX > MX > EB. The adsorptive capacity for C_8_ isomers was determined by thermogravimetric analysis (TGA). Finally, Grand Canonical Monte Carlo (GCMC) calculations were used to simulate the adsorption sites and calculate the adsorption energies.

## 2 Results and Discussion

### 2.1 Crystal Structure of SYUCT-110

Single-crystal X-ray diffraction analysis revealed that SYUCT-110 crystallized in the monoclinic system in the space group, *P*2_1/*C*_. SYUCT-110 is composed of Co metal nodes linked by diimidazole ligands 1,3-beib[1,3-bis(2-ethylimidazol-1-ylethyl) benzene)] and terephthalate anions. The combination of single-crystal X-ray determination and elemental analysis gave the formula SYUCT-110 as Co_2_(1,3-beib)(TPA)_2_. Elemental analysis calcd (%) for C_34_H_30_Co_2_N_4_O_8_: C, 54.10; N, 7.57; H, 4.10; found: C, 54.02; N, 7.85; H, 4.43.

**Figure 1:**
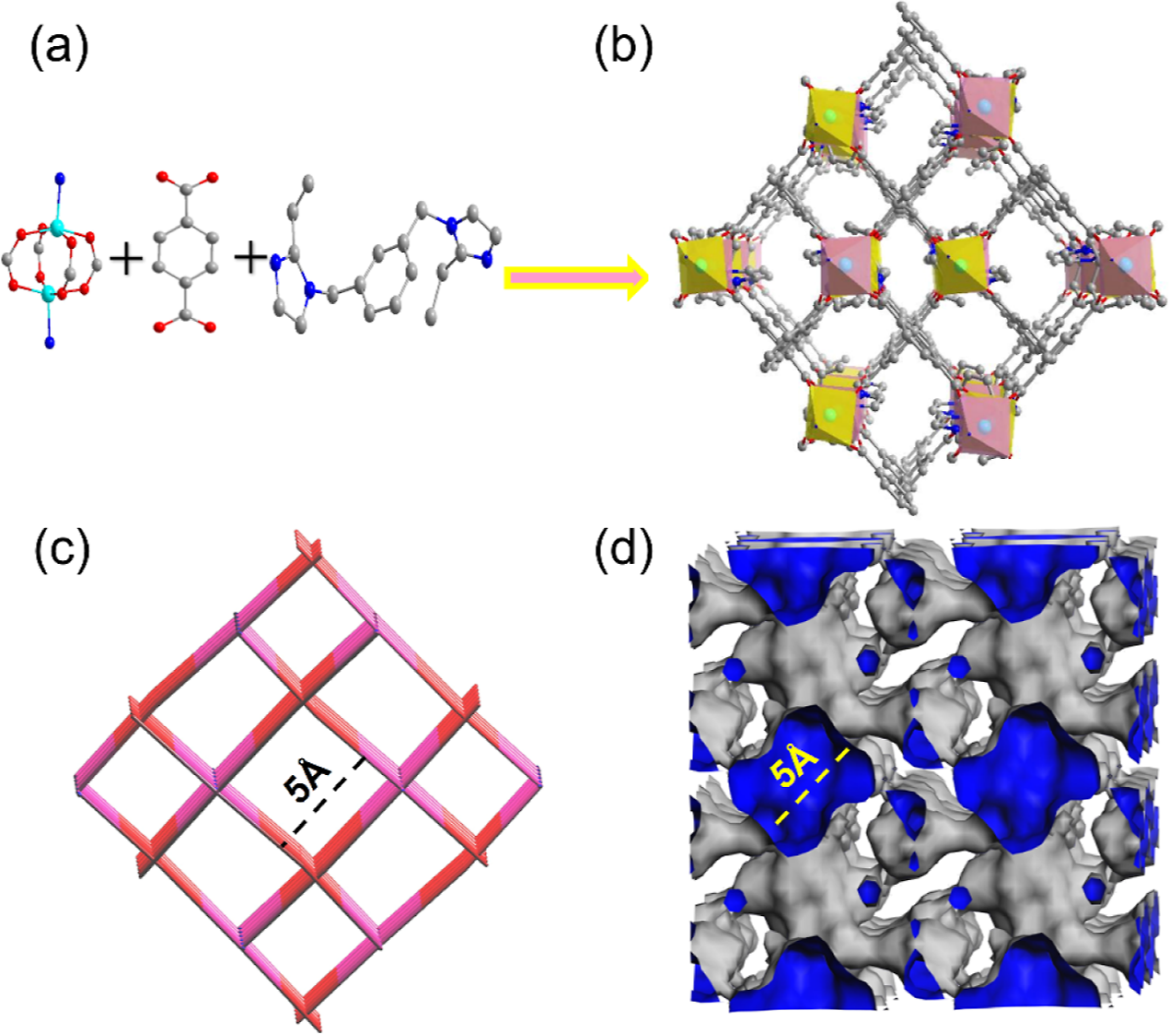
(a) Metal nodes and connectors in SYUCT-110 and (b) SYUCT-110 3D structure diagram in the direction of axis a (turquoise represents cobalt atoms, red represents oxygen atoms, dark gray represents carbon atoms, and hydrogen atoms are not shown). (c) SYUCT-110 topology structure in the direction of axis a (pink represents cobalt metal clusters, red represents terephthalic acid, and blue represents 1,3-beib connectors). (d) Diagram of the SYUCT-110 1D prismatic channel in the direction of axis a (blue is the inside of the channel and gray is the outside of the channel).

Each cobalt cation is coordinated to four oxygen atoms and one nitrogen atom (Figure S1). The Co atoms and terephthalate anions form two-dimensional (2D) layered structures and are further connected by 1,3-beib ligands to form a 3D interpenetrating structure (Figure 1b) with diamond-shaped channels along axis a. More interestingly, due to the interpenetration of the structures, each large rhombic channel is divided into four smaller rhombic channels with side lengths of 5 Å (Figure 1c,d). In addition, Co atoms and terephthalate anions formed 2D-layered pores with a size of 11.4 Å (Figure S9). Due to the large layered pores, there is no separation effect on C_8_ isomers. Therefore, this article studies the 5 Å diamond-shaped channel. Overall, SYUCT-110 possesses 1D diamond-shaped channels and a microporous structure.

### 2.2 Synthesis and Characterization of SYUCT-110

Powder X-ray diffraction (PXRD) of simulated, as-synthesized, and SYUCT-110 crystals was compared (Figure S2). The signal-to-noise ratio in the high-angle region of the PXRD pattern of MOFs makes it impossible to clear the weak peaks.^35^ Therefore, it is necessary to derive position marks for every Miller plane in the plotted range and compare them with simulated and synthesized PXRD patterns.^36−38^ The result shows that some diffraction peaks exhibit shifts and differences in intensity. It is due to the preferred orientation of the crystal. During the growth process of crystals, certain crystal planes exhibit stronger growth trends compared with other crystal planes. The fact is the key that cause changes in the position and intensity of diffraction peaks.^39−41^ Further, the PXRD patterns of SYUCT-110 before and after soaking in MeOH, EtOH, CH_3_CN, DMA, and DMF for 1 day are similar (Figure S3), indicating that the structure remains intact. TGA revealed that the activated form SYUCT-110 has no weight loss <400 °C in N_2_ (Figure S4). The variable temperature PXRD results also indicate that SYUCT-110 can be stabilized to 400 °C (Figure S5). Fourier transform (FTIR) spectroscopy revealed a band at 3444 cm^–1^ consistent with the O–H stretching vibration. This could be caused by the adsorption of some water on the surface of the crystals (Figure S6). The weak bands at 2954 cm^–1^ can be attributed to the C–H stretch of the linker. The strong absorption bands at 1622 cm^–1^ and 1385 cm^–1^ are due to v_asym_(COO) and v_sym_(COO) stretching modes, respectively.^42^ SYUCT-110 was activated in a vacuum oven at 130 °C for 6 h. In order to ensure the correct activation, the activated samples were tested by TGA and PXRD. The TGA test showed that solvent molecules were successfully removed from the activated samples (Figure S4). The PXRD patterns before and after activation were in agreement (Figure S7). To explore the porosity of SYUCT-110, CO_2_ adsorption isotherms were measured (Figure S8). The Brunauer–Emmett–Teller (BET) surface area of SYUCT-110 was evaluated as 249.71 m^2^ g^–1^. And the pore size distribution shows that the 1D prismatic channel size of SYUCT-110 is 4.8 Å. This is consistent with the pore size obtained from the crystal structure. Scanning electron microscopy (SEM) and crystal images under the microscope show that the crystal morphology is block-like (Figure S10). Finally, the proton peaks of the 1,3-beib ligand and terephthalate in SYUCT-110 are shown in Figure S13. The sample contains the proton peak of 1,3-beib ligand, and the carboxyl proton peak in TPA disappears. The results showed that the Co and O in the carboxyl group formed coordination bonds, and the complex was synthesized successfully. The ratio of the two was determined by the integral area of the proton peak. Thus, the ratio of 1,3-beib ligand to terephthalate was 1:2, which matches the crystal structure formula.

### 2.3 Batch Uptake Experiments

**Figure 2:**
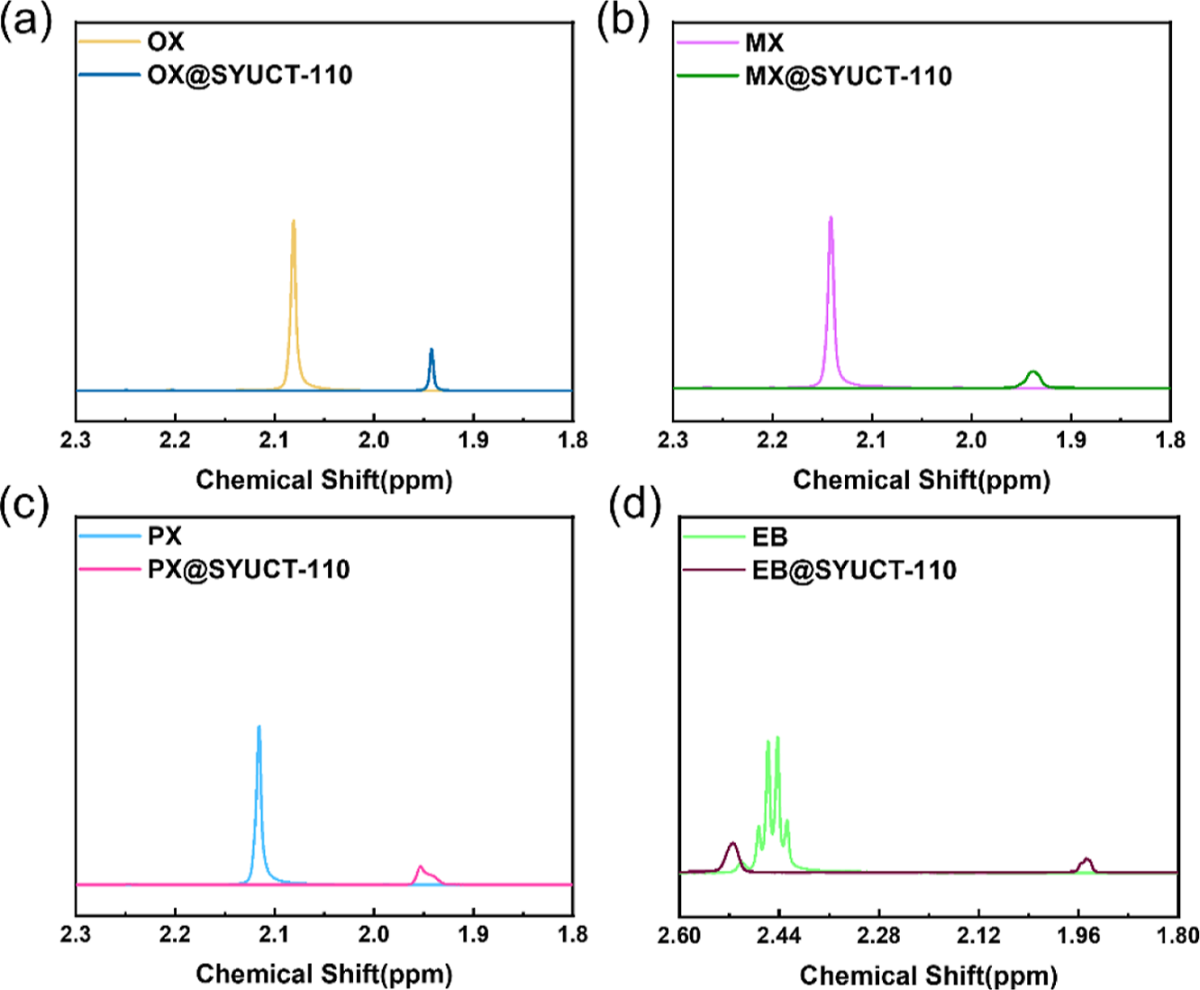
^1^H NMR spectrum of SYUCT-110 was recorded with OX (a), PX (b), MX (c), and EB (d).

In order to study the adsorption and separation performance of SYUCT-110 for C_8_ aromatics, batch single-component absorption experiments were conducted. In single-component batch adsorption experiments, ^1^H NMR spectra of samples soaked with C_8_ aromatics were compared with those of pure C_8_ aromatics and SYUCT-110 (Figures 2 and S13). We found that the ^1^H NMR spectra of samples soaked with C_8_ aromatics showed methyl or ethyl peaks. So, this indicates C_8_ isomer adsorption. We also found that the methyl or ethyl peaks in C_8_ aromatics were shifted after adsorption, which may be due to the difference in sample concentration.^23^

**Figure 3:**
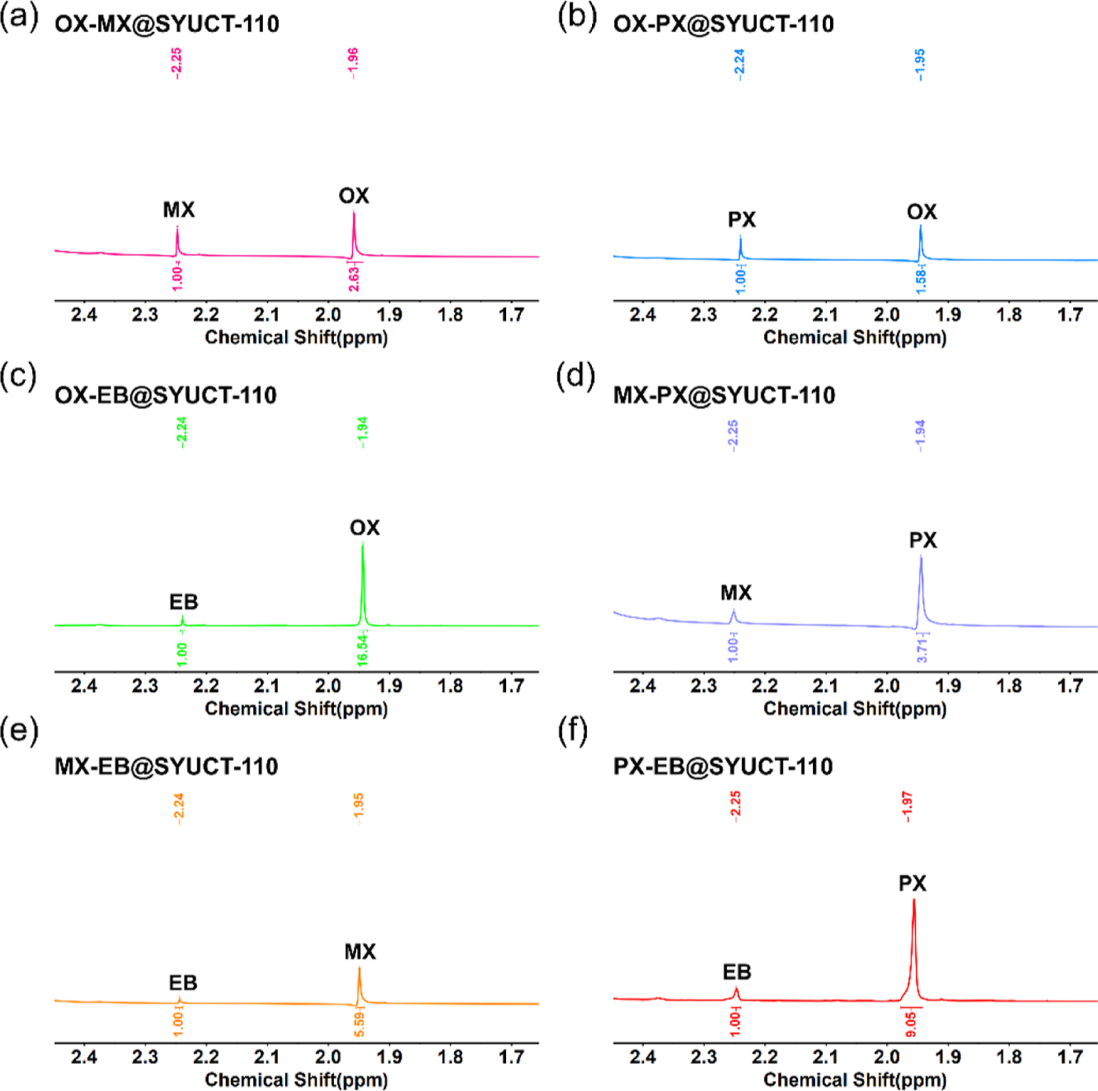
^1^H NMR spectrum of SYUCT-110 for OX/MX selectivity (a), OX/PX selectivity (b), OX/EB selectivity (c), PX/MX selectivity (d), MX/EB selectivity (e), and PX/EB selectivity (f).

To further determine the adsorptive selectivity of SYUCT-110 for C_8_ aromatics, two-component batch adsorption experiments were conducted by soaking the activated sample in equimolar ratio mixtures of C_8_ isomers and testing by ^1^H NMR. The methyl peaks of OX, MX, and PX resonances were located at 2.08, 2.14, and 2.12 ppm, respectively (Figures S14–S16), whereas for EB, methylene protons resonated from 2.43 to 2.47 ppm (Figure S17). ^1^H NMR spectroscopy showed that there were significant differences in the integrated areas and peak intensities of methyl or methylene moieties in C_8_ aromatics (Figure 3a–f).

According to the integral area of ^1^H NMR, selectivity values were found to be 2.63, 1.58, 5.51, 3.71, 1.86, and 3.02 for the X-ray diffraction domains of OX/MX, X-ray diffraction atoms of OX/PX, X-ray diffraction atoms of OX/EB, X-ray diffraction atoms of MX, and X-ray atoms of PX/EB and PX/EB, respectively. To ensure the accuracy of the experiment, we repeated the experiment three times. The average selectivity values were 2.60 (OX/MX), 1.57 (OX/PX), 5.50 (OX/EB), 3.71 (PX/MX), 1.85 (MX/EB), and 3.02 (PX/EB) (Figures S18–S23). SYUCT-110 has a preference for OX over other C_8_ isomers in the following order of selectivity: OX > PX > MX > EB. The value of OX/EB is higher than that of some good-performing adsorbents, including MIL-53(Ga),^43^ Co(dobdc),^6^ MIL-47(V),^44^ MIL-101(Cr),^45^ and Zn(BDC)(Dabco)_0.5_.^46^ The selectivity of OX/PX and OX/MX is comparable to that of other MOFs (Table S2).

**Figure 4:**
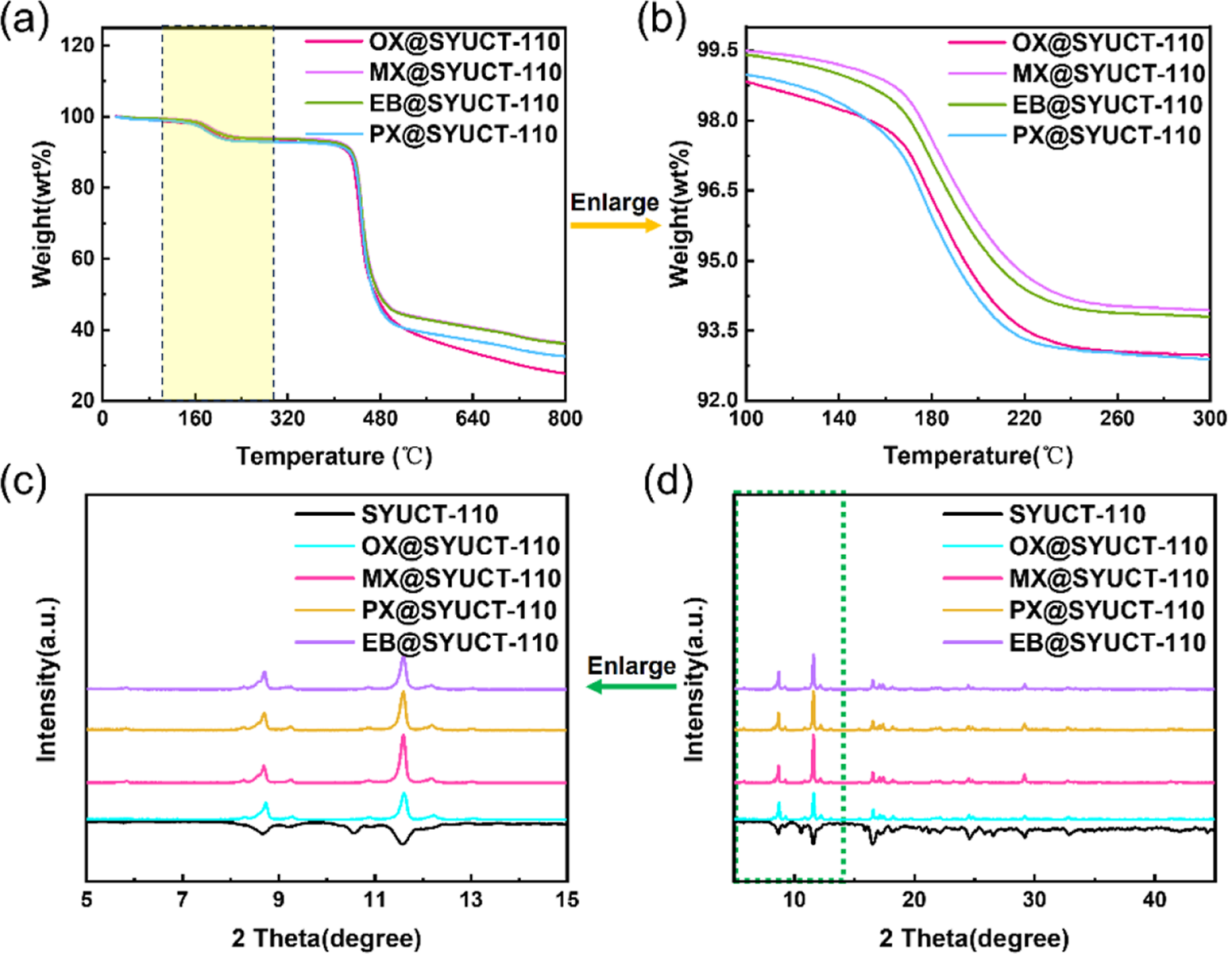
(a,b) TGA diagram of SYUCT-110 after adsorption of C_8_ aromatics. (c,d) PXRD comparison of SYUCT-110 before and after adsorption.

**Figure 5:**
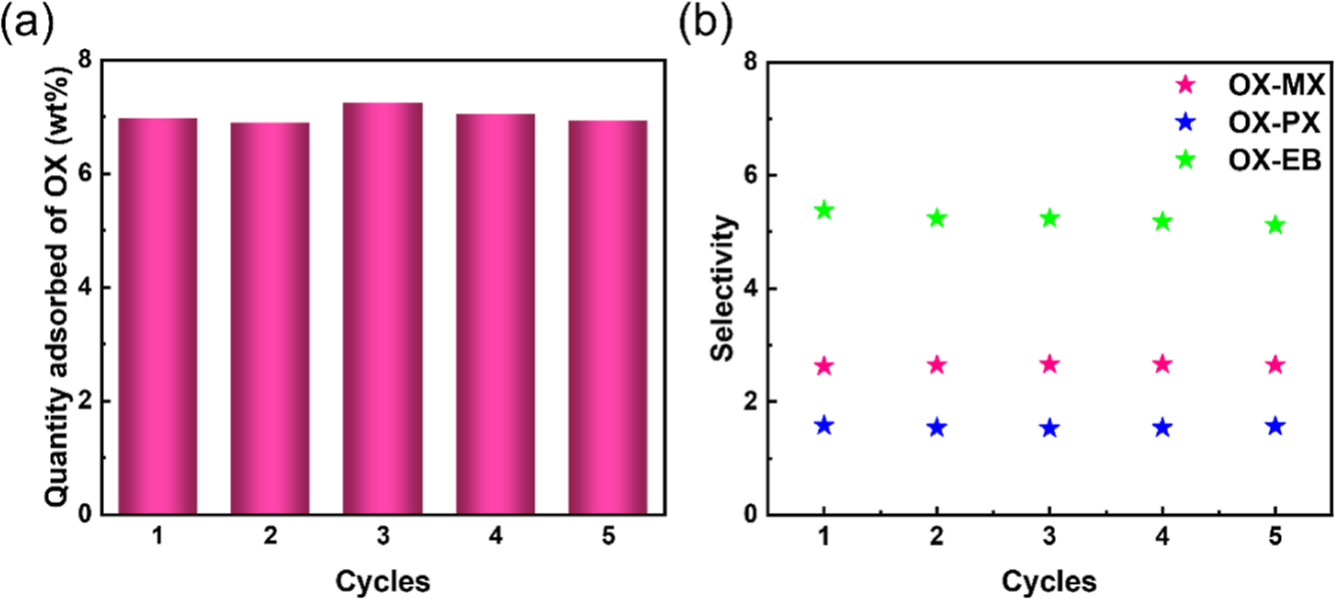
(a) Five consecutive cycles of OX adsorption–desorption on SYUCT-110. (b) Selectivity values of OX/MX, OX/PX, and OX/EB for SYUCT-110 adsorption during five consecutive cycles.

Next, the soaked samples are subjected to TGA and PXRD testing. Data reveal that weight loss occurred at 160 °C consistent with that of C_8_ aromatics (Figures 4a and S24–S27). The adsorption capacities for OX and PX were 7.1%, whereas MX and EB were 6.2% (Figure 4b). These data are consistent with the adsorption selectivity obtained by ^1^H NMR (Table S2). By comparing the PXRD patterns of the samples before and after adsorption, it was found that the peak shape and intensities had changed after adsorption (Figure 4c,d). Although C_8_ isomers have similar kinetic dimensions, the selectivity and adsorption capacity are not the same. This phenomenon might be caused by weak interactions (e.g., π···π, hydrogen bonds, and van der Waals) between adsorbent molecules and adsorbents.^47−49^ In addition, we also evaluated the recyclability and used TGA to determine the adsorption capacity of the C_8_ aromatics. The adsorption capacity of C_8_ aromatics did not decrease significantly after five cycles of adsorption/desorption (Figures 5a and S28–S30). The selectivity during the cycles was monitored by using ^1^H NMR tests. After five cycles, the selectivity values of OX/MX, OX/PX, OX/EB, PX/MX, MX/EB, and PX/EB were 2.65, 1.57, 5.18, 3.59, 1.83, and 3.05, respectively (Figures 5b and S31–S39). The selectivity did not change significantly compared to that before the cycles. The results showed that SYUCT-110 still had good separation performance of C_8_ aromatics. And PXRD patterns of cycled samples were found to be consistent (Figures S40–S43).

### 2.4 GCMC Simulations

**Figure 6:**
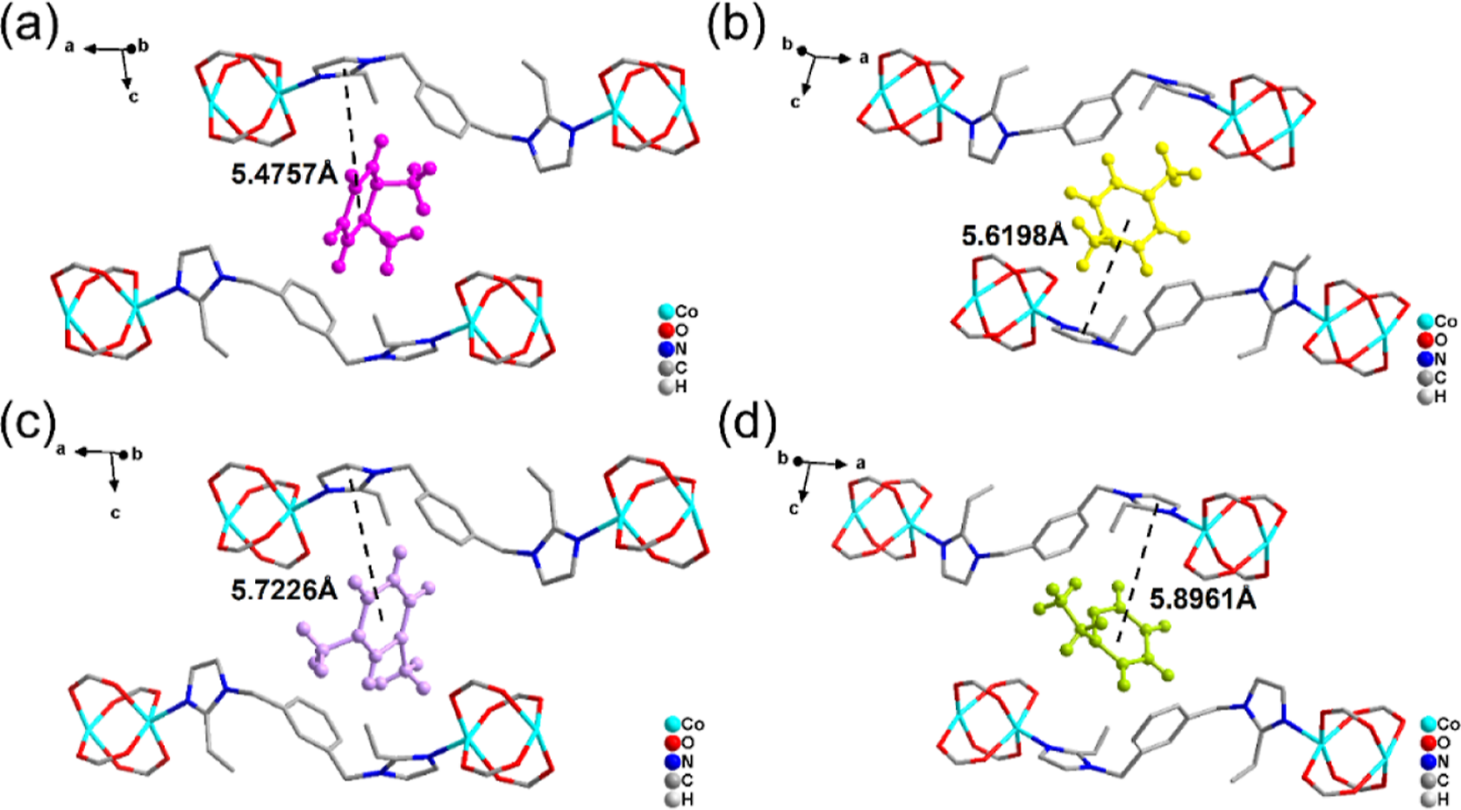
Location of a single OX (a), PX (b), MX (c), and EB (d) molecule in the structure of SYUCT-110.

In order to gain insight into the selectivity of SYUCT-110 for C_8_ aromatics, we used Material Studio software for GCMC simulations. Compared with other C_8_ aromatic hydrocarbons, the π···π interaction distance between OX and the imidazole group in the 1,3-beib connector is the shortest at 5.4757 Å (PX, MX, and EB are 5.6198 Å, 5.7226 Å, and 5.8961 Å, respectively) (Figure 6). This also matches the selectivity obtained in the two-component batch adsorption experiment. In addition, we also found a C–H···π weak interaction between the C atom on the OX benzene ring and the imidazole group, as well as the C–H···N hydrogen weak interaction distances are 4.1802 and 4.2448 Å, respectively (Figure S44). Compared with other isomers (MX, PX, and EB are 4.4744 and 4.3353 Å, 4.5744 and 4.4765 Å, and 4.7546 and 4.6306 Å, respectively), OX has the strongest C–H···π weak interaction and C–H···N hydrogen weak interaction (Table S3). This analysis of the binding sites supports the experimental observations that SYUCT-110 has higher selectivity for OX, which we can now attribute to the stronger π···π interactions, C–H···π interaction, and C–H···N hydrogen bonding.

To further explain the selectivity of OX over other C_8_ aromatics, GCMC simulations were performed to calculate the adsorption energy (*E*_ads_). E_ads_ is calculated as follows^50^
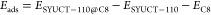
1where *E*_SYUCT-110@C8_ refers to the energy of the sample after adsorption of C_8_ isomer. *E*_SYUCT-110_ refers to the energy of SYUCT-110 before adsorption. *E*_C8_ refers to the energy of a single C_8_ aromatic molecule. The results showed that the adsorption energies of OX, MX, PX, and EB were −286.08, −269.76, −274.56, and −254.40 kJ/mol, respectively (Table S4). These values are on the same order of magnitude as the adsorption energy values of molecules such as benzene, CO_2_, NO_*x*_, and furfuryl alcohol, indicating the accuracy of the calculation results.^51−54^ The adsorption energies of the four isomers are negative, indicating that the isomers are attracted to SYUCT-110. Besides, the adsorption energy of OX is the largest compared to those of other isomers, which indicates the strongest weak interaction between OX and the sample. The results support that SYUCT-110 is more selective to OX than other isomers.

## 3 Conclusions

In this work, the use of an interpenetrating MOF material for the selective separation of C_8_ aromatics is proposed for the first time. The structure contains 1D rhomboid channels. The selectivity of C_8_ aromatics was determined by 2-component batch adsorption experiments. The results showed that the selective order was OX > PX > MX > EB. The selectivity of OX/EB was 5.51. The adsorption capacity of OX was 71 mg/g. In addition, it has good thermal stability (400 °C) and recyclability (can be recycled at least five times). GCMC simulations were used to study the C_8_ adsorption sites, revealing that π···π interactions between imidazole groups and C_8_ aromatic hydrocarbons are the main reason for the observed adsorption selectivity. E_ads_ calculated from GCMC results show that SYUCT-110 and C_8_ isomers attract each other. Compared with other isomers, OX has the highest adsorption energy (−286.06 kJ/mol). It is further clarified that SYUCT-110 has a preference for OX.

## 4 Experimental Section

### 4.1 Materials and Physical Measurements

All chemicals used were purchased from commercial suppliers and were not further purified during use. Crystal data was collected, and crystal structure was determined using X-ray single-crystal diffraction produced by Rigaku Company in Japan at *T* = 100 K. The smartlab9 polycrystalline powder X-ray diffractometer, manufactured by Rigaku, Japan, was used to perform PXRD tests. Crystal appearance was ascertained by the Quanta FEG 450 electron microscope of FEI company and characterized using the Frontier infrared spectrometer produced by PerkinElmer in the United States. TGA was performed on the German NETZSCH SAT4495F5 thermogravimetric analyzer. ^1^H NMR tests were performed using the AVANCE III 500 MHz nuclear magnetic resonance instrument produced by Bruker, Switzerland. The CO_2_ adsorption isotherm was determined using the BSD-660 instrument produced by Beijing Bester.

### 4.2 Synthesis of SYUCT-110

Crystal SYUCT-110 was synthesized by a solvent thermal reaction. 1,3-beib[1,3-Bis(2-ethylimidazol-1-ylethyl)benzene)] (11.0 mg, 0.03 mmol), terephthalic acid (5.0 mg, 0.03 mmol), and Co(NO_3_)_2_·6H_2_O (43.7 mg, 0.15 mmol) with a solvent mixture of 5 mL of *N*,*N*-dimethylacetamide (DMA) and 5 mL of methanol (MeOH) were dissolved in a 25 mL screw-capped glass vial. Then, the vial was capped tightly and heated to 100 °C for 72 h. The blue lumpy crystals of SYUCT-110 were formed and washed three times with fresh MeOH (yield: 48.8%, based on 1,3-beib).

## Supporting Information Available

SEM images, XRD patterns and analyses, FIR spectra, pore size distribution, DTG and TGA analyses, GCMC simulation method, and ^1^H NMR crystallographic data for SYUCT-110 (PDF)
