# Supplementary material for: Selective Separation of C8 Aromatics by an Interpenetrating Metal–Organic Framework Material
Source: Inorg Chem. 2024 Sep 27;63(40):18847–54. doi: 10.1021/acs.inorgchem.4c02969 (PMC11462495; doi:10.1021/acs.inorgchem.4c02969)
Supplement: Supplementary file 1 — ic4c02969_si_001.pdf [file ic4c02969_si_001.pdf]

Supporting Information

Selective separation of C_8 aromatics by an interpenetrating metal-organic framework material

Na Sun,[*a,b] Xue Zhou,[a] Han Yu,[a] Xiuwen Si,[a] Fu Ding,[a] Yaguang Sun*[*a] & Michael J. Zaworotko*[*c]

[*]Email: xtal@ul.ie

[a] Key Laboratory of Inorganic Molecule-Based Chemistry of Liaoning Province, Shenyang University of Chemical Technology, Shenyang 110142, China

[b] School of Materials Science and Engineering National Institute for Advanced Materials TKL of Metal and Molecule-Based Material Chemistry, Nankai University, Tianjin 300350, China

[c] Department of Chemical Sciences and Bernal Institute, University of Limerick, Limerick V94 T9PX, Republic of Ireland

[d] Petrochemical Department, Liaoning Petrochemical College, Jinzhou 121001, China

Experimental Section

Materials and Methods

All reagents and solvents used are commercially available and require no further purification. 1, 3-bis(2-ethylimidazol-1-ylethyl) was purchased from Jinan Henghua Technology Co., LTD. Methanol and DMA were purchased from Tianjin Damao Chemical Reagent Factory, and $Co(NO_3)_2\cdot 6H_2O$ was purchased from Sinopod Group Chemical Reagent Co., LTD. Powder X-ray diffraction (PXRD) was performed using

smartlab9 polycrystalline powder X-ray diffraction in the 2θ range of 3-80°. The simulated pattern is generated by Mercury program and single crystal diffraction data. The thermogravimetric analysis of samples was performed on the Nitsch SAT449F5 thermogravimetric analyzer produced in Germany under nitrogen protection, at a temperature range of 30 to 800 ° C and a heating rate of 10 ° C min^{-1}.

Batch Uptake Experiments

In order to ensure that the adsorption reached saturation and the experimental results were accurate, we carried out batch adsorption experiments for 48h.[1-3] In the batch absorption experiment, 10mg activated SYUCT-110 powder was immersed in four equimolar single-component C_8 aromatic stock solution for 48h. Under ambient conditions (about 25 ° C), the sample that has reached adsorption saturation was filtered on filter paper in the fume hood and air-dried. Due to the volatility of C_8 aromatics, the C_8 isomers attached to the surface of the sample can be removed after 3-5 minutes. After, 5mg of the sample was put into the nuclear magnetic tube, 5μL concentrated hydrochloric acid was added to release the C_8 aromatics molecules in the structure, and 450μL d6-DMSO was added to dissolve the samples.[4-8] Four pure C_8 aromatic isomers of 50μL were respectively loaded into nuclear magnetic tubes for ^1H NMR measurement using the same steps to ensure that no other factors interfered with their chemical shifts. Finally, ^1H NMR measurements were performed using a 500MHz nuclear magnetic resonance spectrometer.

C_8 aromatics selectivity calculation

The adsorption selectivity of SYUCT-110 can be determined by the relative peak areas as follows：

$$s_{ij} = \frac{x_i y_j}{x_j y_i} \quad (1)$$ [1]

s_{ij} refers to the selectivity of component i with respect to component j, x_i and x_j refer to the molar fractions of i and j in the adsorption phase, and y_i and y_j refer to the molar fractions of i and j in the liquid phase. For an equimolar binary liquid system, selectivity can be simplified as:

$$s_{ij} = \frac{x_i}{x_j} \quad (2)$$

The x_i/x_j ratio can be obtained by the relative integrated area of the ^1H NMR spectra of methyl or methylene groups corresponding to C_8 aromatics. When both components i and j are xylene isomers, s_{ij} can be defined as:

$$s_{ij} = \frac{x_i}{x_j} = \frac{q_i}{q_j} \quad (3)$$

q_i and q_j refer to the relatively integrated area of the methyl peak corresponding to the xylene isomer in the ^1H NMR. When j is ethylbenzene, s_{ij} can be defined as:

$$s_{ij} = \frac{x_i}{x_j} = \frac{q_i}{3q_j} \quad (4)$$

q_i refers to the relative integral area of methyl group (including 6 H) in the xylene isomer, while q_j refers to the relative integral area of ethylbenzene methylene group (including 2H).

Recyclability test

The activated SYUCT-110 was immersed in four kinds of C_8 isomer solution for 48h at room temperature. The adsorbed samples are then heated in a vacuum oven at 130℃ for 6h to complete the desorption process. Some samples were tested by PXRD to

determine whether the skeleton was intact after an adsorption-desorption process. Then the samples were soaked in C_8 aromatics stock solution for adsorption process. After 48h, the samples were taken out and put on the filter paper in the fume hood and air dried. After 3-5 minutes, the C_8 aromatics attached to the sample surface can be removed. The samples were then tested by TGA and ^1H NMR respectively to determine the adsorption capacity and selectivity after one adsorption and desorption. The above process is repeated five times to obtain the adsorption capacity, selectivity and PXRD of the samples after the five cycles of adsorption-desorption.

Grand Canonical Monte Carlo (GCMC) Simulations

The adsorption sites were determined by GCMC simulation[9, 10].The adsorption sites of SYUCT-110 for C_8 aromatics were calculated by using the adsorption module in Materials Studio software. Using the Sorption module, locate the task. GCMC simulation uses 1×1×1 cell, and the simulation sets 1×10^5 maximum loading step, 1×10^5 production step and 4 temperature cycles. COMPASS force field and Ewald&Group electrostatic field are selected. Select the metropolis method, fine quality. Van der waals select atom based. Truncation Select cubic spline. Cutoff distance set to 12.4Å and spline width set to 1Å.

Next, GCMC simulation was used to calculate the adsorption energy (E_{ads}). The CASTEP module in Materials Studio software was used for calculation. Perform geometric optimization on SYUCT-110. During geometric optimization set max iterations to 100, max SCF cycles to 100, and run in parallel to 12. After optimization, the energy of the four isomers and SYUCT-110 before and after adsorption were

calculated respectively. In the process of calculating energy set max iterations to 100, max SCF cycles to 100, and run in parallel to 4.

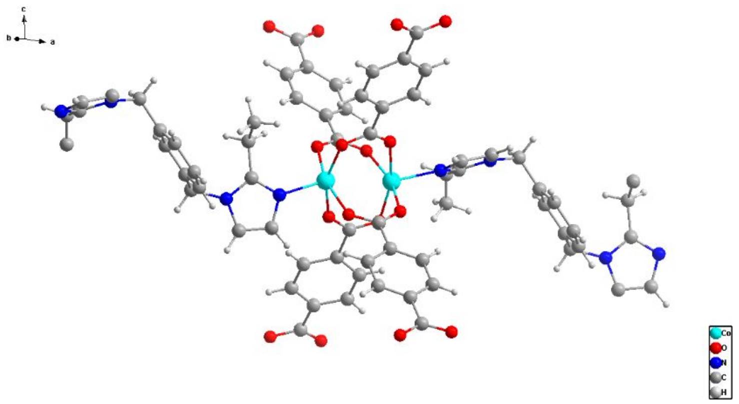

Figure S1. Local structure of SYUCT-110 detailing the metal coordination environments.

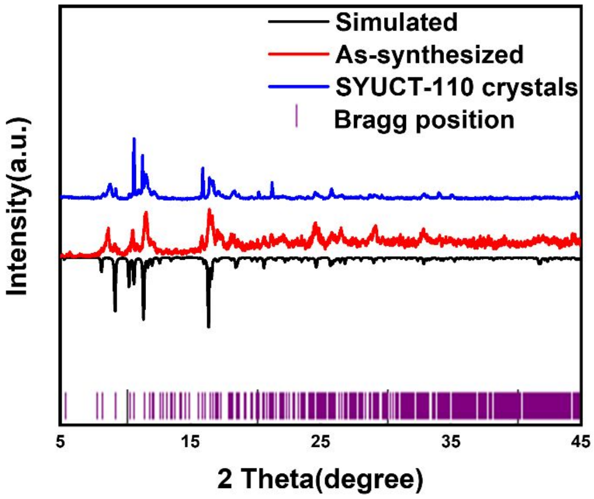

Figure S2. PXRD pattern of SYUCT-110.

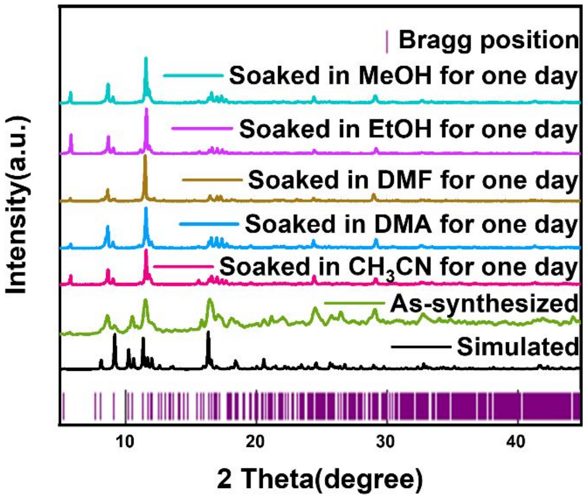

Figure S3. PXRD pattern of different SYUCT-110 samples.

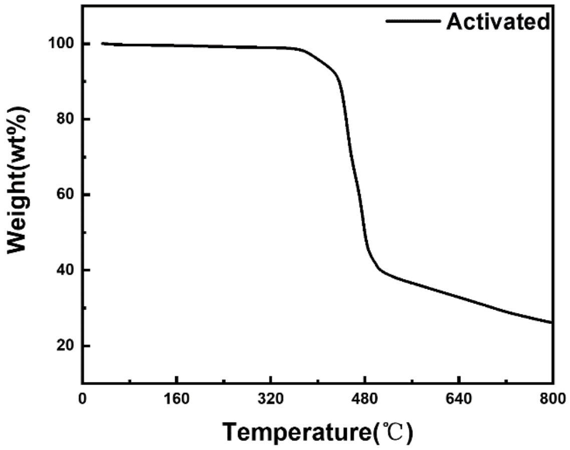

Figure S4. TGA pattern of activated SYUCT-110.

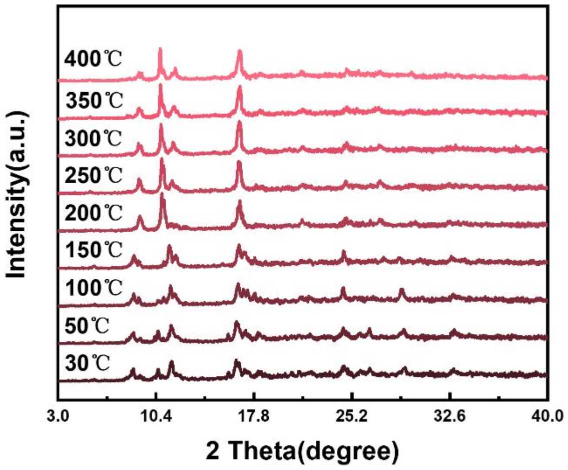

Figure S5. Variable-temperature PXRD pattern of SYUCT-110.

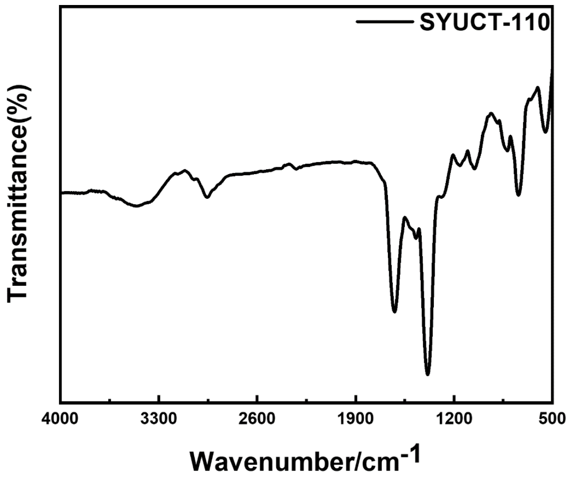

Figure S6. FTIR pattern of SYUCT-110.

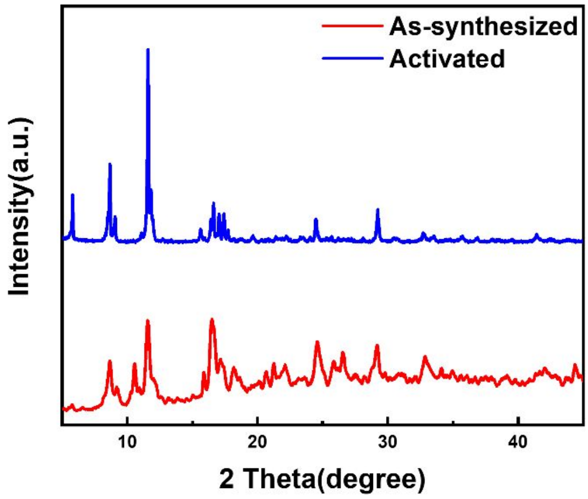

Figure S7. PXRD pattern of activated SYUCT-110.

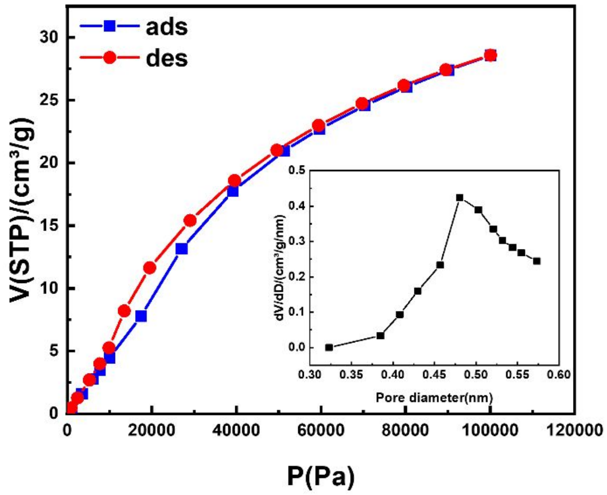

Figure S8. CO_2 adsorption/desorption isotherm at 298K and pore size distribution for SYUCT-110.

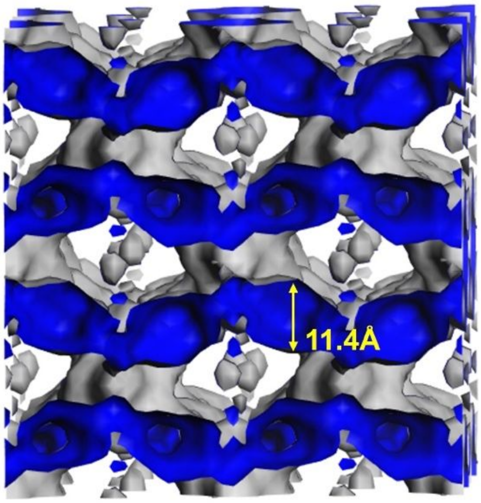

Figure S9. Interlayer pore size in SYUCT-110.

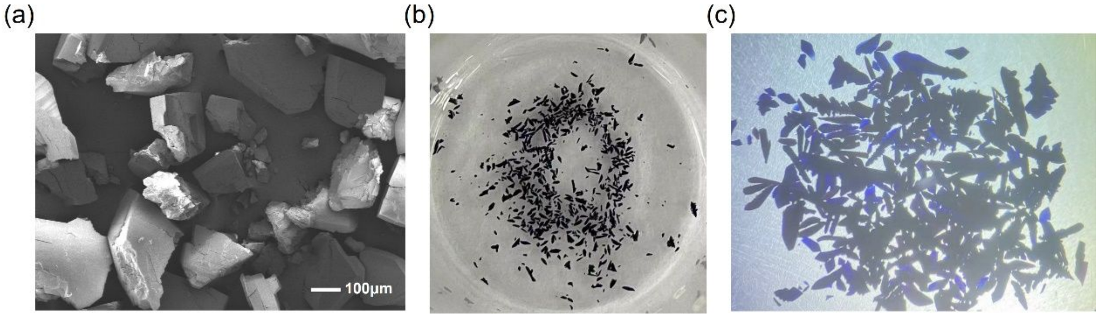

Figure S10. (a) Scanning electron microscope image of SYUCT-110. (b) Crystal morphology of SYUCT-110 under environmental conditions. (c) SYUCT-110 observed under a microscope.

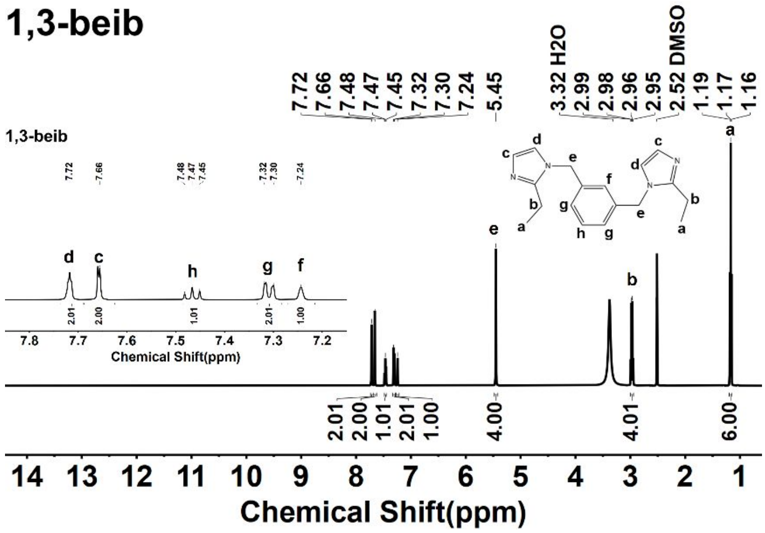

Figure S11. ^{1}H NMR spectrum of ligands 1,3-beib.

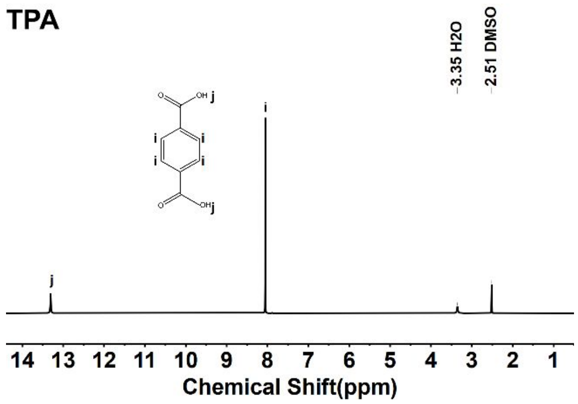

Figure S12. ^{1}H NMR spectrum of 1,4-dicarboxybenzene.

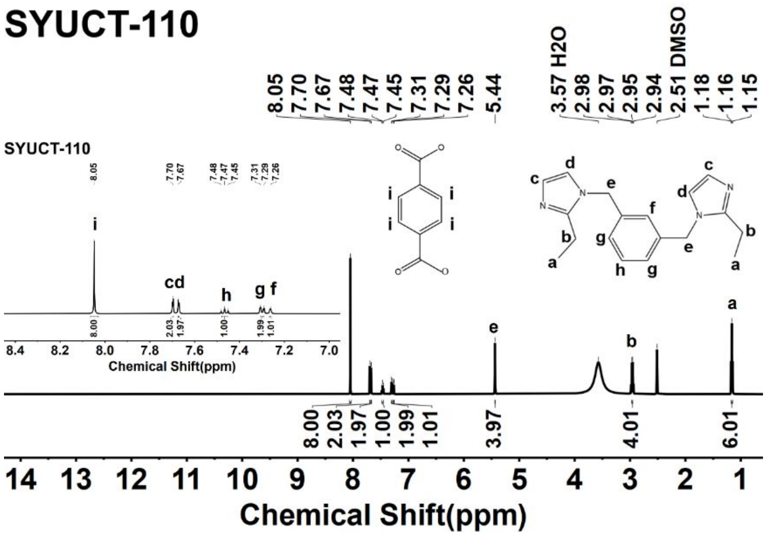

Figure S13. ^{1}H NMR spectrum of SYUCT-110.

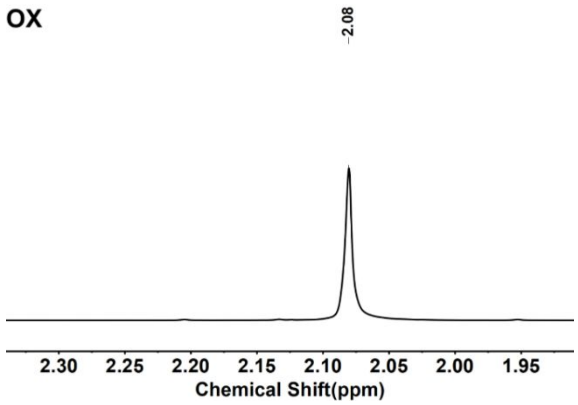

Figure S14. ^{1}H NMR spectrum of o-xylene.

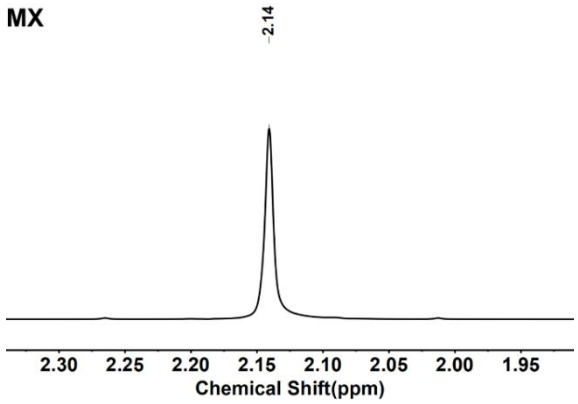

Figure S15. ^{1}H NMR spectrum of m-xylene.

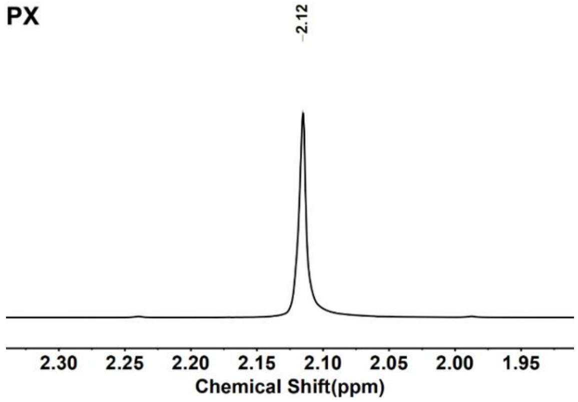

Figure S16. ^{1}H NMR spectrum of p-xylene.

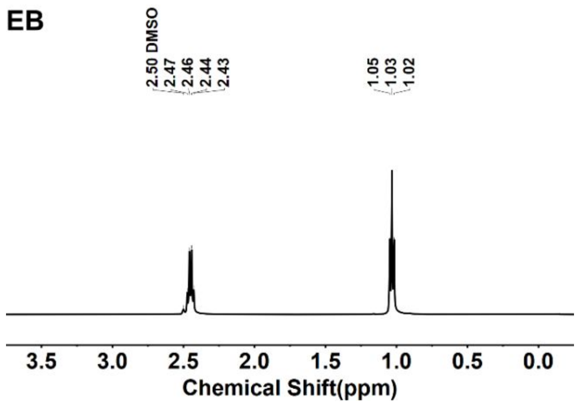

Figure S17. ^{1}H NMR spectrum of ethylbenzene.

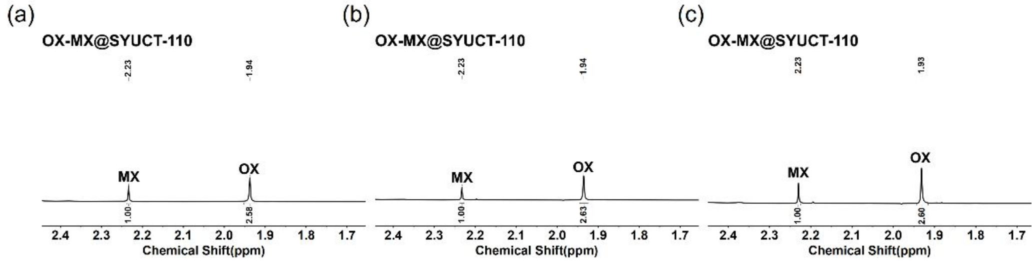

Figure S18. (a-c) ¹H NMR spectrum of SYUCT-110 for OX/MX selectivity.

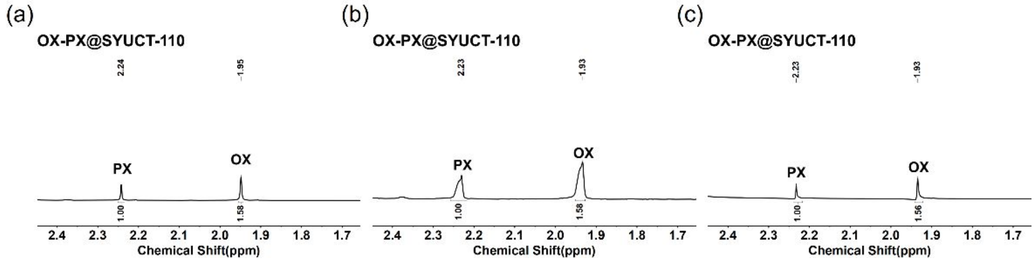

Figure S19. (a-c) ¹H NMR spectrum of SYUCT-110 for OX/PX selectivity.

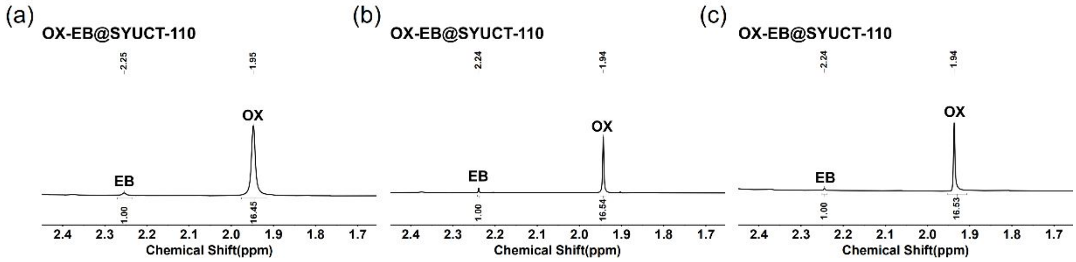

Figure S20. (a-c) ¹H NMR spectrum of SYUCT-110 for OX/EB selectivity.

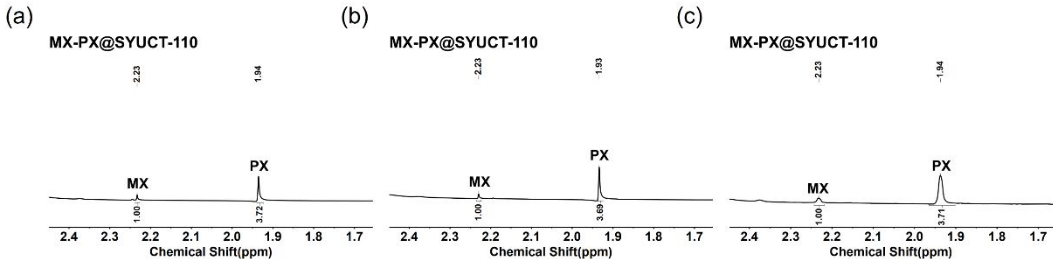

Figure S21. (a-c) ¹H NMR spectrum of SYUCT-110 for PX/MX selectivity.

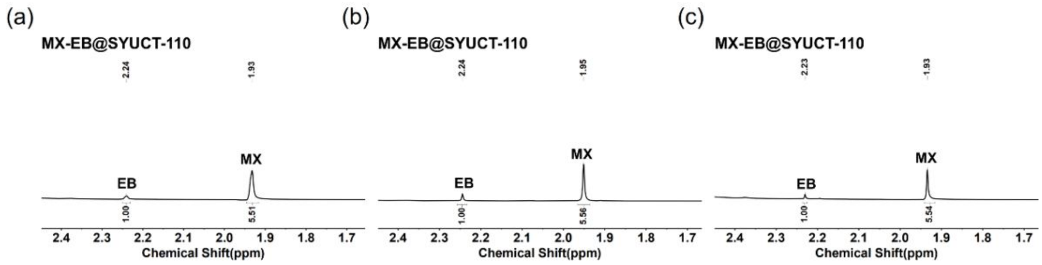

Figure S22. (a-c) ^{1}H NMR spectrum of SYUCT-110 for MX/EB selectivity.

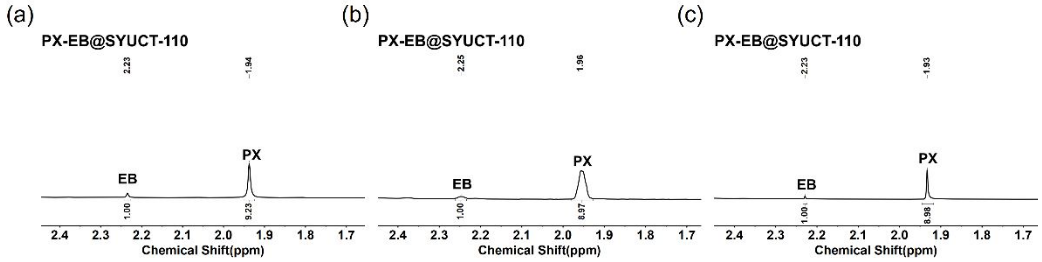

Figure S23. (a-c) ^{1}H NMR spectrum of SYUCT-110 for PX/EB selectivity.

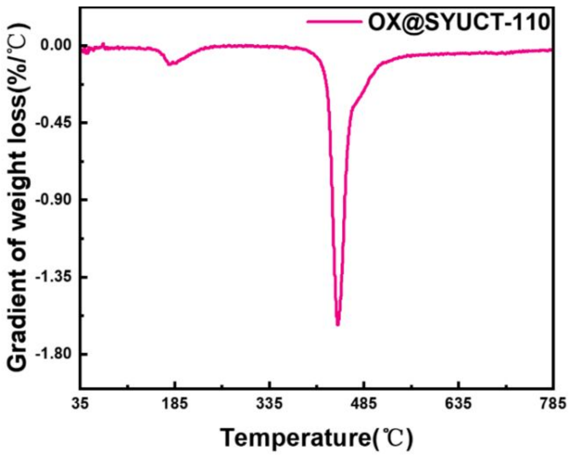

Figure S24. DTG analyses of OX@SYUCT-110.

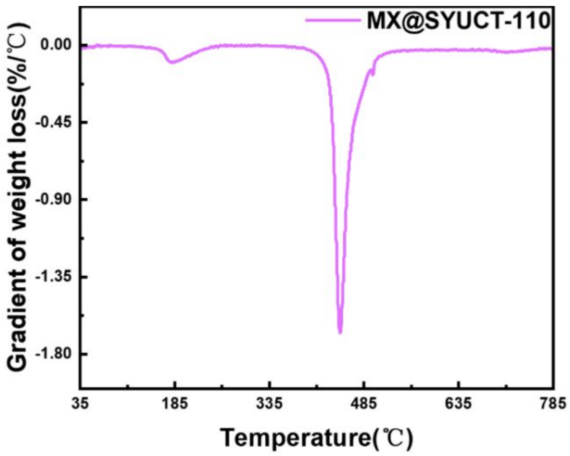

Figure S25. DTG analyses of MX@SYUCT-110.

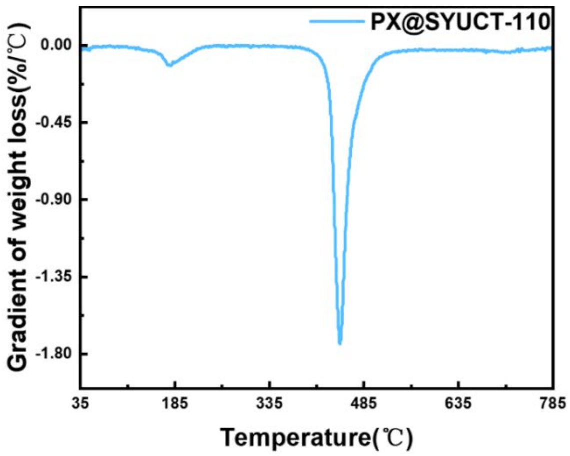

Figure S26. DTG analyses of PX@SYUCT-110.

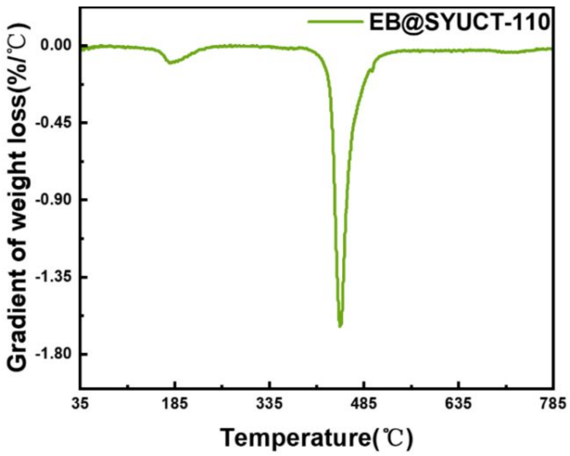

Figure S27. DTG analyses of EB@SYUCT-110.

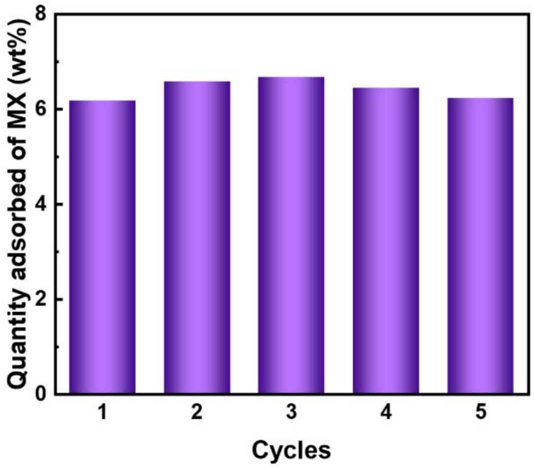

Figure S28. Five consecutive cycles of MX adsorption-desorption on SYUCT-110.

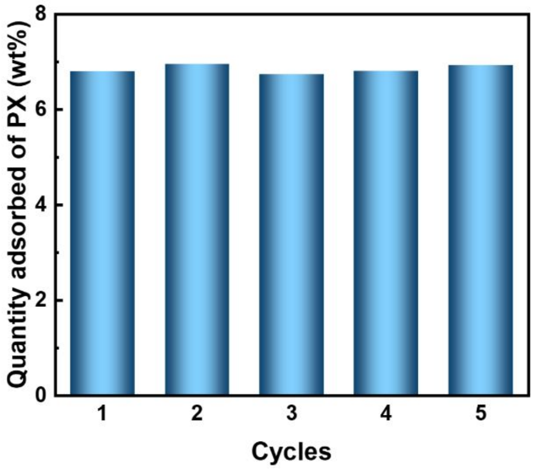

Figure S29. Five consecutive cycles of PX adsorption-desorption on SYUCT-110.

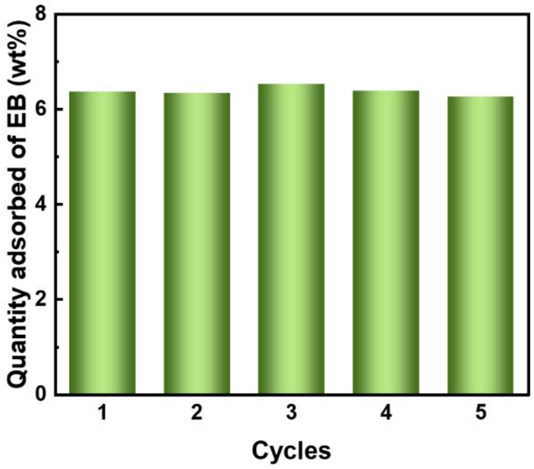

Figure S30. Five consecutive cycles of EB adsorption-desorption on SYUCT-110.

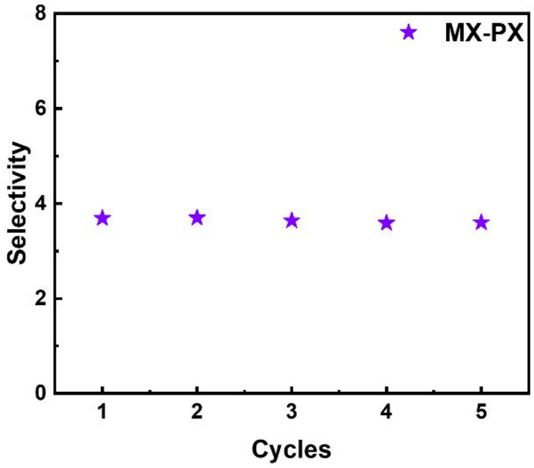

Figure S31. The selectivity values of MX/PX for SYUCT-110 adsorption during five consecutive cycles.

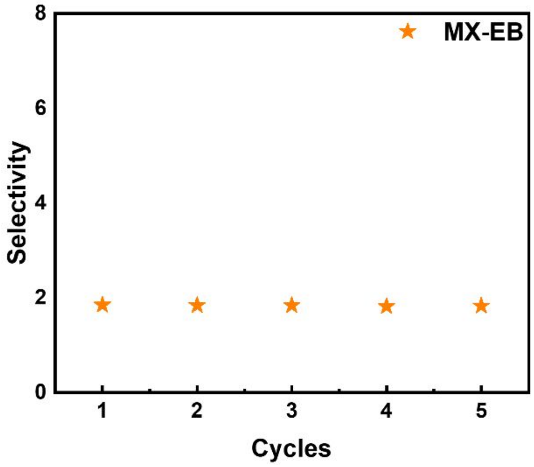

Figure S32. The selectivity values of MX/EB for SYUCT-110 adsorption during five consecutive cycles.

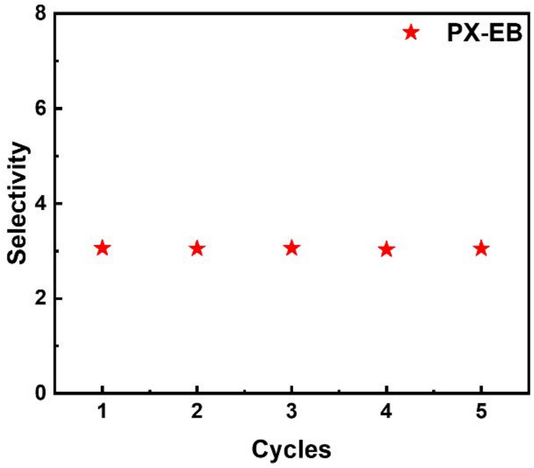

Figure S33. The selectivity values of PX/EB for SYUCT-110 adsorption during five consecutive cycles.

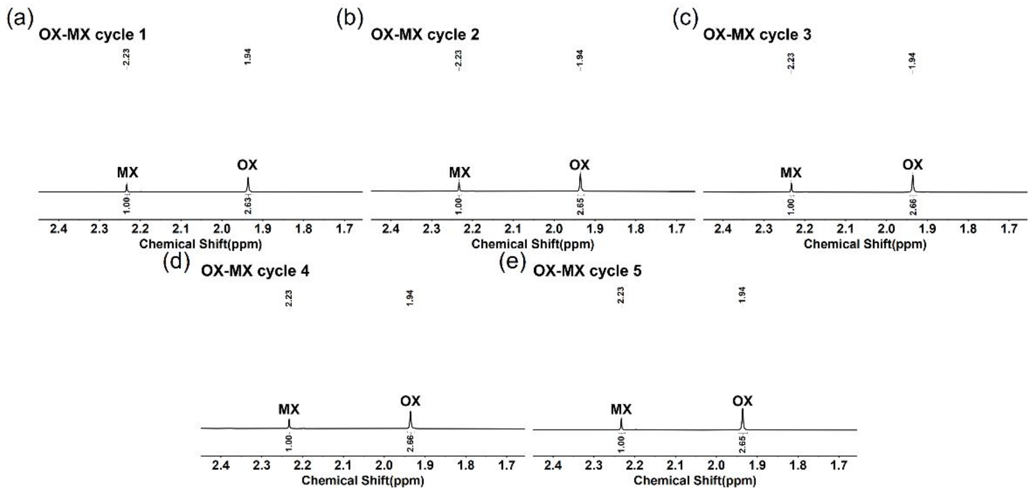

Figure S34. (a-e) ^{1}H NMR spectrum of OX-MX@SYUCT-110 adsorption during five consecutive cycles.

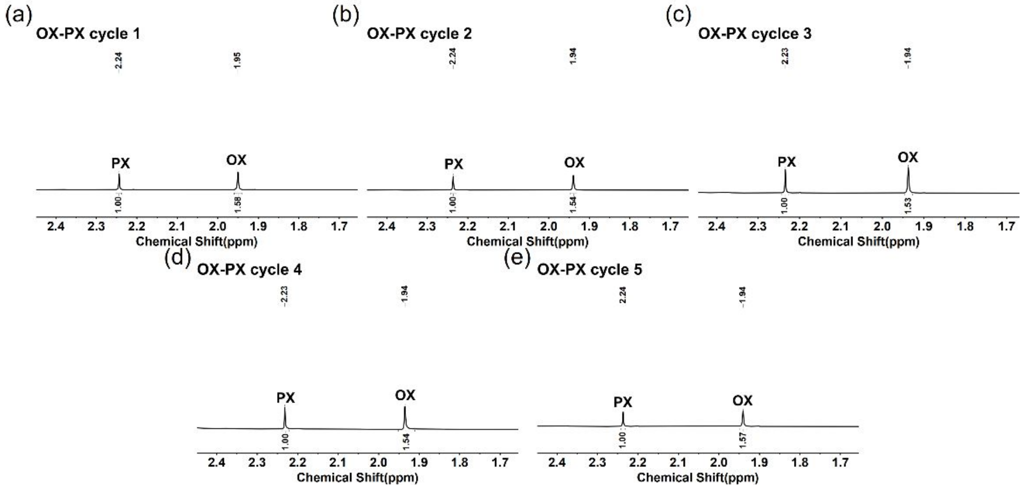

Figure S35. (a-e) ^{1}H NMR spectrum of OX-PX@SYUCT-110 adsorption during five consecutive cycles.

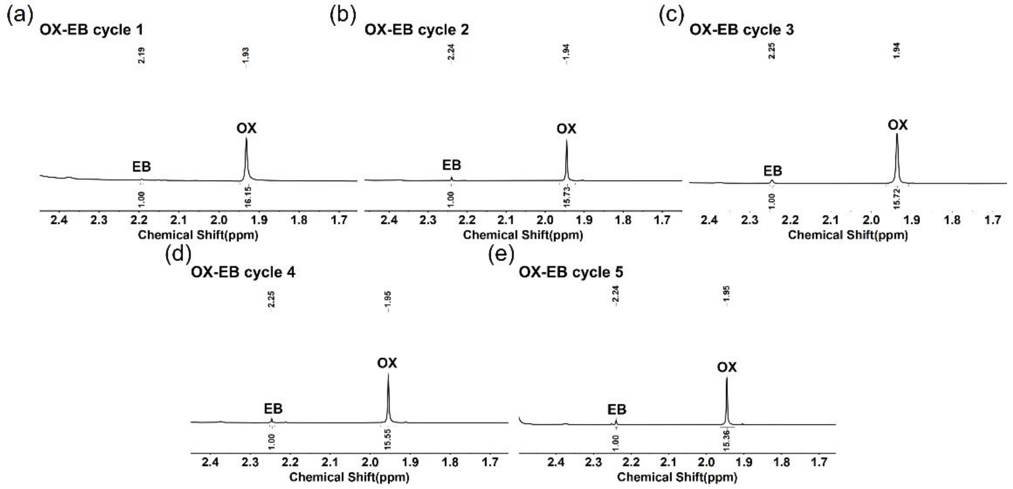

Figure S36. (a-e) ^{1}H NMR spectrum of OX-EB@SYUCT-110 adsorption during five consecutive cycles.

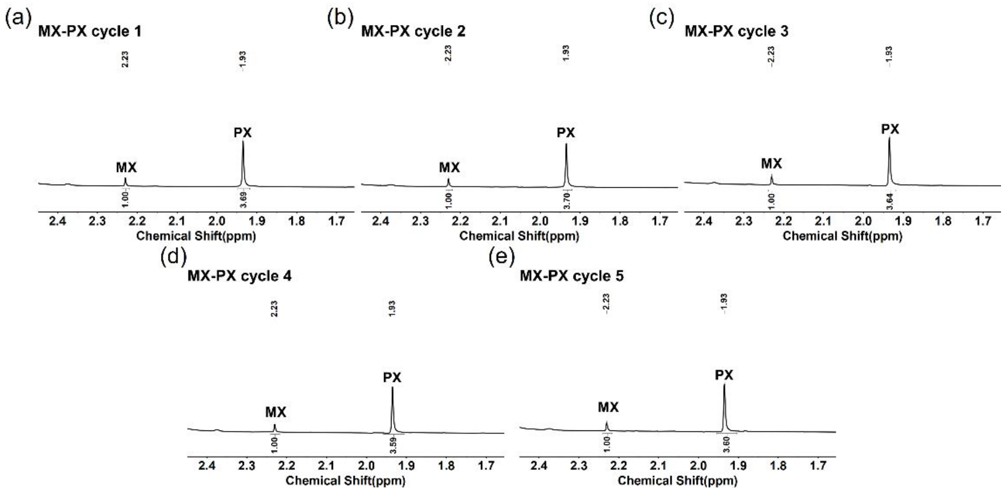

Figure S37. (a-e) ^{1}H NMR spectrum of MX-PX@SYUCT-110 adsorption during five consecutive cycles.

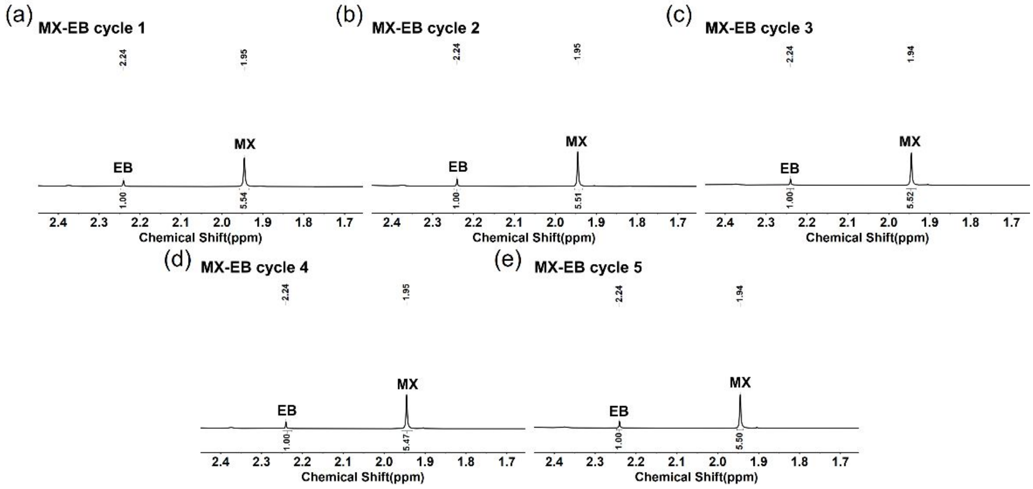

Figure S38. (a-e) ^{1}H NMR spectrum of MX-EB@SYUCT-110 adsorption during five consecutive cycles.

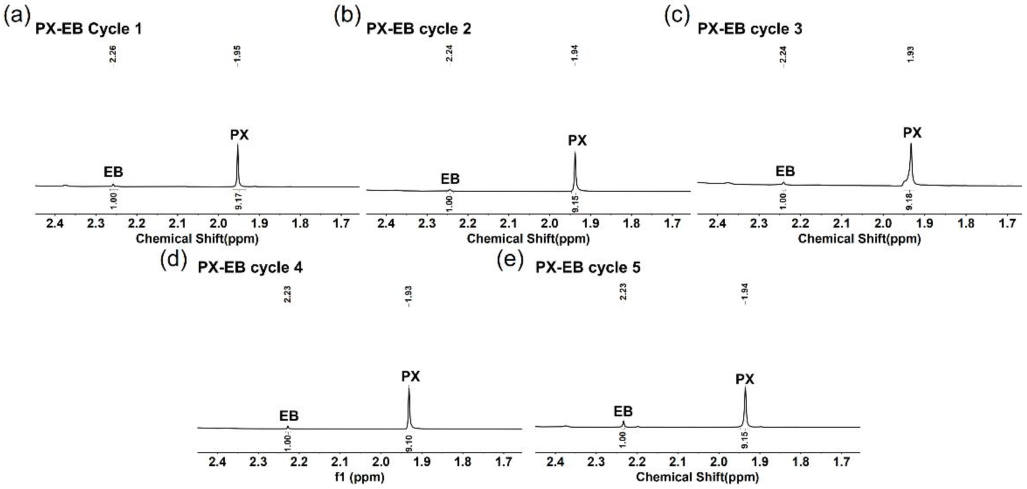

Figure S39. (a-e) ^{1}H NMR spectrum of PX-EB@SYUCT-110 adsorption during five consecutive cycles.

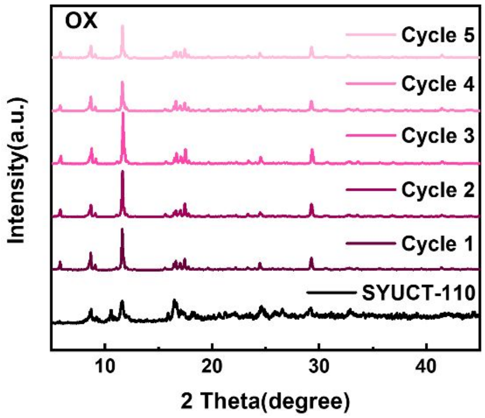

Figure S40. PXRD of OX adsorption-desorption on SYUCT-110 for five consecutive cycles.

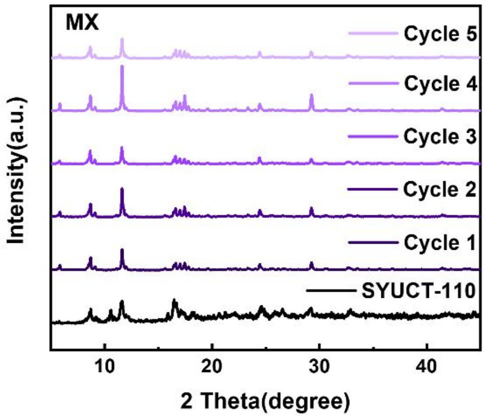

Figure S41. PXRD pattern of MX adsorption-desorption on SYUCT-110 for five consecutive cycles.

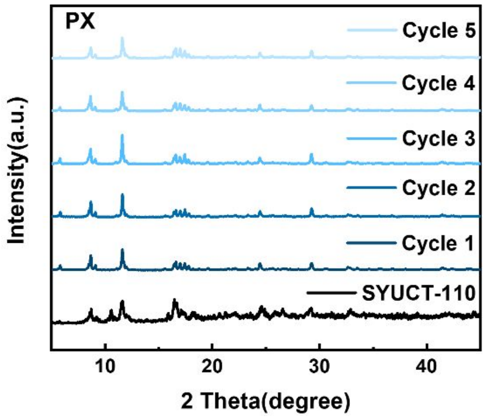

Figure S42. PXRD pattern of PX adsorption-desorption on SYUCT-110 for five consecutive cycles.

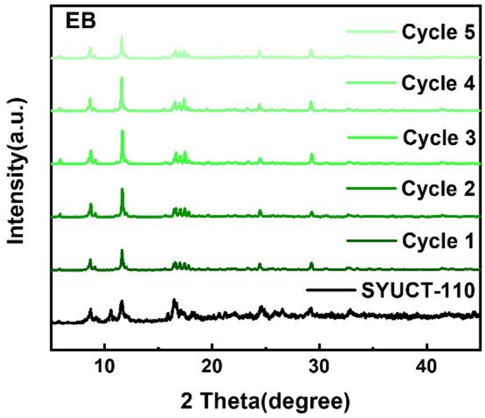

Figure S43. PXRD pattern of EB adsorption-desorption on SYUCT-110 for five consecutive cycles.

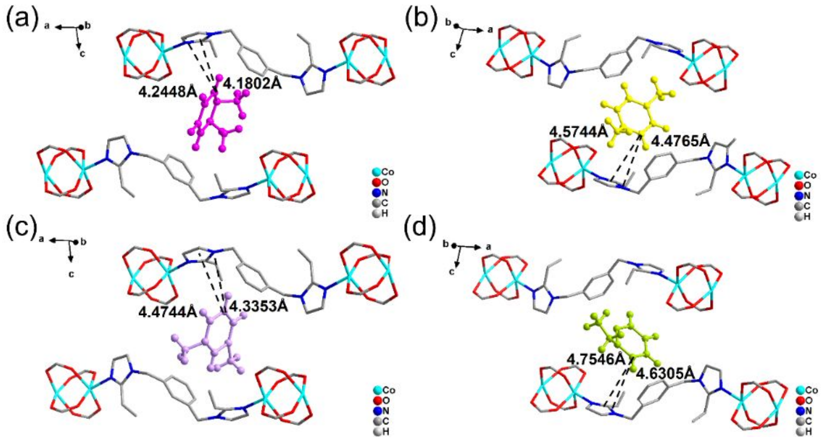

Figure S44. SYUCT-110 interacts with C-H... π and C-H... N of OX (a), MX (b), PX (C), and EB (d).

Table S1. Crystallographic data of SYUCT-110.

Identification code	SYUCT-110
CCDC number	2347496
Empirical formula	$C_{34}H_{30}Co_2N_4O_8$
Formula weight	740.48
Temperature/K	297
Crystal system	Monoclinic
Space group	$P2_1/c$
a/Å	17.0332(2)

b/Å	15.5687(2)
c/Å	15.2594(2)
α/°	90
β/°	99.3890(10)
γ/°	90
Volume/Å^3	3992.35(9)
Z	4
ρ_{calc}g/cm^3	1.232
μ/mm^{-1}	6.913
F(000)	1520.0
Radiation	CuK$_\alpha$ (λ = 1.54184)
Reflections collected	21304
Independent reflections	7003 [R_{int} = 0.0196, R_{sigma} = 0.0234]
Data/restraints/parameters	7003/1/436
Goodness-of-fit on F^2	1.108
Final R indexes	R_1 = 0.0430
[I>=2σ (I)]	wR_2 = 0.1335
Final R indexes	R_1 = 0.0529
[all data]	wR_2 = 0.1391

Table S2. The summary of adsorption selectivity of C_8 aromatics by adsorbents.

Adsorbents	Selectivity					
	OX/MX	OX/PX	OX/EB	PX/MX	MX/EB	PX/EB

SYUCT-110	2.68	1.58	5.50	3.70	1.85	3.02
MIL-47(Ⅴ)[11]	1.17	1.01	1.39	2.07	1.41	1.83
MIL-101(Cr)[12]	1.50	1.60	1.40	1/1.10	1/1.10	NG.
MOF-5[13]	NG.	NG.	1.96	NG.	2.34	4.14
CAU-13[14]	1.90	1.50	NG.	1.30	NG.	NG.
Zn(BDC)(Dabco)$_{0.5}$[15]	1.12	1.88	1.62	1/1.25	1.15	1/1.15
Co_2(dobdc)[16]	2.50	3.90	1.21	1/1.60	1/2.05	1/3.21
MIL-53(Cr)[17]	2.80	3.70	4.90	1/1.40	NG.	NG.
MIL-53(Ga)[17]	2.50	3.30	4.70	1/1.40	NG.	NG.
MIL-53(Al)[17]	5.10	5.20	8.20	NG.	NG.	NG.
ZUL-C3[4]	7.71	21.8	7.81	NG.	NG.	NG.
Sql-1-Co-NCS[1]	7.50	9.60	60.10	1/1.30	3.80	7.30
NU-2000[18]	NG.	1/20.00	NG.	3.90	NG.	NG.
CD-MOF-1[19]	3.90	14.60	NG.	1/11.20	NG.	NG.
HIAM-201[20]	1/5.80	1/24.40	NG.	4.20	NG.	NG.
Mn-dhbq[21]	1/47.20	1/76.90	NG.	18.20	NG.	NG.
DUT-8(Cu)[22]	NG.	1/8.30	NG.	12.50	NG.	8.80
MFM-300(In)[23]	1/2.70	1.40	NG.	1/3.80	NG.	NG.
Zn-ETTOB[24]	1/3.50	1/6.20	1/13.10	3.90	1/3.30	1.20
HKUST-1[25]	1/0.92	1/0.83	NG.	0.89	NG.	NG.
CPO-27-Ni[25]	1/0.60	1/0.30	1/0.50	NG.	NG.	NG.
SIFSIX-1-Cu[26]	1.10	2.70	NG.	1/2.30.	NG.	NG.
AZO-Cage[27]	NG.	1/15.60	NG.	10.90	NG.	9.70
EtP5[28]	NG.	1/1.87	NG.	2.08	NG.	NG.
EtP6[28]	NG.	1/14.28	NG.	10.20	NG.	NG.
P[4]Q[1]L[29]	NG.	1/22.71	NG.	9.54	NG.	20.67
AgLClO4(M)[30]	1/3.93.	1/24.00	NG.	6.19	NG.	10.36
TPBDαI[31]	1/54.10	NG.	NG.	50.60	NG.	47.3
MCF-50[32]	1/1.30	1/1.60	NG.	1.30	NG.	NG.
Ce(HTCPB)[33]	1/1.20	1/5.70	NG.	4.60	NG.	NG.
NaYmicrocrystalline[34]	1/2.38	NG.	NG.	1/2.62	5.93	NG.
NaYnanocrystalline[34]	1/3.16	NG.	NG.	1/2.90	6.88	NG.
H/ZMS-5[35]	NG.	1/6.782	NG.	24.986	NG.	6.76

Li/ZMS-5[35]	NG.	1/5.987	NG.	8.213	NG.	33.977
Na/ZMS-5[35]	NG.	1/5.451	NG.	6.721	NG.	2.008
K/ZMS-5[35]	NG.	1/3.906	NG.	3.977	NG.	1.101
BaXnanosize[36]	NG.	1/2.819	NG.	7.191	NG.	3.745
KaXnanosize[37]	NG.	1/2.43	NG.	5.36	NG.	3.22
COF 1[38]	2.00	NG.	NG.	1/1.30	NG.	1.40
BaYzeolite[39]	NG.	1/4.00	NG.	3.90	NG.	1.80

Note: NG. refers to not given. For each material, only the best/highest values of selectivity were selected for comparison.

Table S3. Interaction distance between SYUCT-110 and C_8 aromatics.

	O-xylene	M-xylene	P-xylene	Ethylbenzene

$\pi_{Ar}\ldots\pi_{imid}$(Å)	5.4757	5.7226	5.6198	5.8961
$\pi_{Ar}\ldots\pi_{Ar'}$(Å)	5.1377	4.7882	5.1168	5.2260
$\pi_{Ar}\ldots\pi_{Ar''}$(Å)	5.7932	5.7317	5.9133	5.4861
$C_{Ar}H\ldots\pi_{imid}$(Å)	4.1802	4.4744	4.5744	4.7546
$C_{Ar}H\ldots\pi_{Ar'}$(Å)	3.6996	3.3841	3.6395	3.7691
$C_{Ar}H\ldots\pi_{Ar''}$(Å)	5.2954	5.1902	5.3180	4.7530
$C_{Ar}H\ldots N$(Å)	4.2448	4.3353	4.4765	4.6305
$C_{Ar}H\ldots O$(Å)	4.7205	4.4892	4.5296	4.8170
$C_{me/et}H\ldots\pi_{imid}$(Å)	4.5292	4.2437	3.6472	6.1353
$C_{me/et}H\ldots\pi_{Ar'}$(Å)	4.2116	4.1999	4.2434	4.3590
$C_{me/et}H\ldots\pi_{Ar''}$(Å)	4.9541	4.6353	5.1675	4.4895
$C_{me/et}H\ldots N$(Å)	4.6678	4.4122	3.7357	5.1036
$C_{me/et}H\ldots O$(Å)	4.6569	4.3505	3.8320	5.7071
$C_{Ar}\ldots Co$(Å)	5.0721	5.6022	4.9925	5.4126
$C_{me/et}\ldots Co$(Å)	5.4715	5.2581	4.5800	6.4734

Note: Ar refers to the benzene ring in C_8 aromatics; imid refers to the imidazole group in 1,3-beib; Ar' refers to the benzene ring in 1, 3-beib; Ar" refers to the benzene ring in terephthalic acid; me/et refers to the methyl or ethyl group in C_8 aromatics.

Table S4. The adsorption energy of SYUCT-110 for C_8 aromatics isomers.

	$E_{SYUCT-110@C8}$(ev)	E_{C8}(ev)	$E_{SYUCT-110}$(ev)	E_{ads}(ev)	E_{ads}(kJ/mol)
OX	-51934.58	-1416.45	-50515.15	-2.98	-286.08
MX	-51933.87	-1415.91	-50515.15	-2.81	-269.76
PX	-51934.56	-1416.55	-50515.15	-2.86	-274.56
EB	-51933.57	-1415.77	-50515.15	-2.65	-254.40

References

1. Wang, S. Q.; Mukherjee, S.; Patyk-Kaźmierczak, E.; Darwish, S.; Bajpai, A.; Yang, Q. Y.; Zaworotko, M. J., Highly Selective, High-Capacity Separation of O-Xylene from C_8 Aromatics by a Switching Adsorbent Layered Material. *Angew. Chem. Int. Ed.* **2019**, *58*, 6630-6634.

2. Huang, W.; Jiang, J.; Wu, D.; Xu, J.; Xue, B.; Kirillov, A. M., A Highly Stable Nanotubular MOF Rotator for Selective Adsorption of Benzene and Separation of Xylene Isomers. *Inorg. Chem.* **2015**, *54*, 10524-10526.

3. Kumar, N.; Wang, S.-Q.; Mukherjee, S.; Bezrukov, A. A.; Patyk-Kaźmierczak, E.; O'Nolan, D.; Kumar, A.; Yu, M.-H.; Chang, Z.; Bu, X.-H.; Zaworotko, M. J., Crystal Engineering of a Rectangular Sql Coordination Network to Enable Xylenes Selectivity over Ethylbenzene. *Chem. Sci.* **2020**, *11*, 6889-6895.

4. Zhou, J.; Ke, T.; Song, Y.; Cai, H.; Wang, Z. a.; Chen, L.; Xu, Q.; Zhang, Z.; Bao, Z.; Ren, Q.; Yang, Q., Highly Efficient Separation of C_8 Aromatic Isomers by Rationally Designed Nonaromatic Metal-Organic Frameworks. *J. Am. Chem. Soc.* **2022**, *144*, 21417-21424.

5. Lee, J.; Kim, Y.; Son, Y.; Kim, H.; Nam Choi, Y.; D'Alessandro, D.; Chandra Rao, P.; Yoon, M., Breathing-Assisted Selective Adsorption of C_8 Alkyl Aromatics in Zn-Based Metal-Organic Frameworks. *Chem. - Eur. J.* 2021, *27*, 14851-14857.

6. Chattopadhyay, K.; Mandal, M.; Maiti, D. K., Smart Metal-Organic Frameworks for Biotechnological Applications: A Mini-Review. *ACS Appl. Bio Mater.* 2021, *4*, 8159-8171.

7. Fei, H.; Shin, J.; Meng, Y. S.; Adelhardt, M.; Sutter, J.; Meyer, K.; Cohen, S. M., Reusable Oxidation Catalysis Using Metal-Monocatecholato Species in a Robust Metal-Organic Framework. *J. Am. Chem. Soc.* 2014, *136*, 4965-4973.

8. Wang, T. C.; Vermeulen, N. A.; Kim, I. S.; Martinson, A. B. F.; Stoddart, J. F.; Hupp, J. T.; Farha, O. K., Scalable Synthesis and Post-Modification of a Mesoporous Metal-Organic Framework Called NU-1000. *Nat. Protoc.* 2015, *11*, 149-162.

9. Xiang, F.; Zhang, H.; Yang, Y.; Li, L.; Que, Z.; Chen, L.; Yuan, Z.; Chen, S.; Yao, Z.; Fu, J.; Xiang, S.; Chen, B.; Zhang, Z., Tetranuclear Cu^{II} Cluster as the Ten Node Building Unit for the Construction of a Metal-Organic Framework for Efficient C_2H_2/CO_2 Separation. *Angew. Chem. Int. Ed.* 2023, *62,* e202300638.

10. Ma, L.-N.; Wang, Z.-H.; Zhang, L.; Hou, L.; Wang, Y.-Y.; Zhu, Z., Extraordinary Separation of Acetylene-Containing Mixtures in a Honeycomb Calcium-Based MOF with Multiple Active Sites. *ACS Appl. Mater. Interfaces.* 2023, *15*, 2971-2978.

11. Finsy, V.; Verelst, H.; Alaerts, L.; De Vos, D.; Jacobs, P. A.; Baron, G. V.; Denayer, J. F. M., Pore-Filling-Dependent Selectivity Effects in the Vapor-Phase Separation of Xylene Isomers on the Metal-Organic Framework MIL-47. *J. Am. Chem. Soc.* **2008**, *130*, 7110-7118.

12. Gu, Z. Y.; Yan, X. P., Metal-Organic Framework MIL-101 for High-Resolution Gas-Chromatographic Separation of Xylene Isomers and Ethylbenzene. *Angew. Chem. Int. Ed.* **2010**, *49*, 1477-1480.

13. Gu, Z.-Y.; Jiang, D.-Q.; Wang, H.-F.; Cui, X.-Y.; Yan, X.-P., Adsorption and Separation of Xylene Isomers and Ethylbenzene on Two Zn-Terephthalate Metal-Organic Frameworks. *J. Phys. Chem. C* **2010**, *114*, 311-316.

14. Niekiel, F.; Lannoeye, J.; Reinsch, H.; Munn, A. S.; Heerwig, A.; Zizak, I.; Kaskel, S.; Walton, R. I.; de Vos, D.; Llewellyn, P.; Lieb, A.; Maurin, G.; Stock, N., Conformation-Controlled Sorption Properties and Breathing of the Aliphatic Al-MOF [Al(OH)(CDC)]. *Inorg. Chem.* **2014**, *53*, 4610-4620.

15. Nicolau, M. P. M.; Barcia, P. S.; Gallegos, J. M.; Silva, J. A. C.; Rodrigues, A. E.; Chen, B., Single- and Multicomponent Vapor-Phase Adsorption of Xylene Isomers and Ethylbenzene in a Microporous Metal-Organic Framework. *J. Phys. Chem. C* **2009**, *113*, 13173-13179.

16. Gonzalez, M. I.; Kapelewski, M. T.; Bloch, E. D.; Milner, P. J.; Reed, D. A.; Hudson, M. R.; Mason, J. A.; Barin, G.; Brown, C. M.; Long, J. R., Separation of

Xylene Isomers through Multiple Metal Site Interactions in Metal-Organic Frameworks. *J. Am. Chem. Soc.* **2018**, *140*, 3412-3422.

17. Agrawal, M.; Bhattacharyya, S.; Huang, Y.; Jayachandrababu, K. C.; Murdock, C. R.; Bentley, J. A.; Rivas-Cardona, A.; Mertens, M. M.; Walton, K. S.; Sholl, D. S.; Nair, S., Liquid-Phase Multicomponent Adsorption and Separation of Xylene Mixtures by Flexible MIL-53 Adsorbents. *J. Phys. Chem. C.* **2017**, *122*, 386-397.

18. Idrees, K. B.; Li, Z.; Xie, H.; Kirlikovali, K. O.; Kazem-Rostami, M.; Wang, X.; Wang, X.; Tai, T.-Y.; Islamoglu, T.; Stoddart, J. F.; Snurr, R. Q.; Farha, O. K., Separation of Aromatic Hydrocarbons in Porous Materials. *J. Am. Chem. Soc.* **2022**, *144*, 12212-12218.

19. Chen, L.; Zhu, D. D.; Ji, G. J.; Yuan, S.; Qian, J. F.; He, M. Y.; Chen, Q.; Zhang, Z. H., Efficient Adsorption Separation of Xylene Isomers Using a Facilely Fabricated Cyclodextrin-Based Metal-Organic Framework. *J. Chem. Technol. Biotechnol.* **2018**, *93*, 2898-2905.

20. Lin, Y.; Zhang, J.; Pandey, H.; Dong, X.; Gong, Q.; Wang, H.; Yu, L.; Zhou, K.; Yu, W.; Huang, X.; Thonhauser, T.; Han, Y.; Li, J., Efficient Separation of Xylene Isomers by Using a Robust Calcium-Based Metal-Organic Framework through a Synergetic Thermodynamically and Kinetically Controlled Mechanism. *J. Mater. Chem. A.* **2021**, *9*, 26202-26207.

21. Li, L.; Guo, L.; Olson, D. H.; Xian, S.; Zhang, Z.; Yang, Q.; Wu, K.; Yang, Y.; Bao, Z.; Ren, Q.; Li, J., Discrimination of Xylene Isomers in a Stacked Coordination Polymer. *Science.* **2022**, *377*, 335-339.

22. Kim, S.-I.; Lee, S.; Chung, Y. G.; Bae, Y.-S., The Origin of P-Xylene Selectivity in a Dabco Pillar-Layered Metal-Organic Framework: A Combined Experimental and Computational Investigation. *ACS Appl. Mater. Interfaces.* **2019**, *11*, 31227-31236.

23. Li, X.; Wang, J.; Bai, N.; Zhang, X.; Han, X.; da Silva, I.; Morris, C. G.; Xu, S.; Wilary, D. M.; Sun, Y.; Cheng, Y.; Murray, C. A.; Tang, C. C.; Frogley, M. D.; Cinque, G.; Lowe, T.; Zhang, H.; Ramirez-Cuesta, A. J.; Thomas, K. M.; Bolton, L. W.; Yang, S.; Schröder, M., Refinement of Pore Size at Sub-Angstrom Precision in Robust Metal-Organic Frameworks for Separation of Xylenes. *Nat. Commun.* **2020**, *11,* 4280.

24. Deng, X.; Deng, M.; Li, Y.-L.; Liu, Z.; Liu, T.-F.; Chen, X.; Cai, S.; Liu, B.; Li, J.; Lv, D.; Li, J.; Yuan, W., A Zinc-Octacarboxylate MOF with an Unusual (6, 8)-Connected ocu Topology for High-Capacity Adsorptive Separation of C_8 Alkylaromatics. *Chem. Eng. J.* **2023**, *474*, 145694-145701.

25. Peralta, D.; Barthelet, K.; Pérez-Pellitero, J.; Chizallet, C.; Chaplais, G.; Simon-Masseron, A.; Pirngruber, G. D., Adsorption and Separation of Xylene Isomers: CPO-27-Ni Vs HKUST-1 Vs NaY. *J. Phys. Chem. C.* **2012**, *116*, 21844-21855.

26. Yang, L.; Liu, H.; Xing, J.; Yuan, D.; Xu, Y.; Liu, Z., Separation of Xylene Isomers in the Anion-Pillared Square Grid Material SIFSIX-1-Cu. *Chem. - Eur. J.* **2021**, *27*, 6187-6190.

27. Moosa, B.; Alimi, L. O.; Shkurenko, A.; Fakim, A.; Bhatt, P. M.; Zhang, G.; Eddaoudi, M.; Khashab, N. M., A Polymorphic Azobenzene Cage for Energy-Efficient and Highly Selective P-Xylene Separation. *Angew. Chem. Int. Ed.* **2020**, *59*, 21367-21371.

28. Jie, K.; Liu, M.; Zhou, Y.; Little, M. A.; Pulido, A.; Chong, S. Y.; Stephenson, A.; Hughes, A. R.; Sakakibara, F.; Ogoshi, T.; Blanc, F.; Day, G. M.; Huang, F.; Cooper, A. I., Near-Ideal Xylene Selectivity in Adaptive Molecular Pillar[N]Arene Crystals. *J. Am. Chem. Soc.* **2018**, *140,* 6921-6930.

29. Zhang, G.; Zhang, K.; Lou, X.-Y.; Li, X.; Song, C.-L.; Huan, W.; Yang, Y.-W., Separation of P-Xylene from C_8 Alkylaromatics by Nonporous Adaptive Crystals of Leggero Pillar[4]Arene[1]Quinone. *ACS Mater. Lett.* **2023**, *6,* 446-451.

30. Sun, N.; Wang, S.-Q.; Zou, R.; Cui, W.-G.; Zhang, A.; Zhang, T.; Li, Q.; Zhuang, Z.-Z.; Zhang, Y.-H.; Xu, J.; Zaworotko, M. J.; Bu, X.-H., Benchmark Selectivity P-Xylene Separation by a Non-Porous Molecular Solid through Liquid or Vapor Extraction. *Chem. Sci.* **2019**, *10*, 8850-8854.

31. Rahmani, M.; Matos, C. R. M. O.; Wang, S.-Q.; Bezrukov, A. A.; Eaby, A. C.; Sensharma, D.; Hjiej-Andaloussi, Y.; Vandichel, M.; Zaworotko, M. J., Highly

Selective P-Xylene Separation from Mixtures of C_8 Aromatics by a Nonporous Molecular Apohost. *J. Am. Chem. Soc.* **2023**, *145*, 27316-27324.

32. Lin, J.-M.; He, C.-T.; Liao, P.-Q.; Lin, R.-B.; Zhang, J.-P., Structural, Energetic and Dynamic Insights into the Abnormal Xylene Separation Behavior of Hierarchical Porous Crystal. *Sci. Rep.* **2015**, *5,* 11537.

33. Warren, J. E.; Perkins, C. G.; Jelfs, K. E.; Boldrin, P.; Chater, P. A.; Miller, G. J.; Manning, T. D.; Briggs, M. E.; Stylianou, K. C.; Claridge, J. B.; Rosseinsky, M. J., Shape Selectivity by Guest-Driven Restructuring of a Porous Material. *Angew. Chem. Int. Ed.* **2014**, *53*, 4592-4596.

34. Rasouli, M.; Yaghobi, N.; Chitsazan, S.; Sayyar, M. H., Effect of Nanocrystalline Zeolite Na-Y on Meta-Xylene Separation. *Microporous Mesoporous Mater.* **2012**, *152*, 141-147.

35. Rasouli, M.; Yaghobi, N.; Chitsazan, S.; Sayyar, M. H., Influence of Monovalent Cations Ion-Exchange on Zeolite ZSM-5 in Separation of Para-Xylene from Xylene Mixture. *Microporous Mesoporous Mater.* **2012**, *150*, 47-54.

36. Rasouli, M.; Yaghobi, N.; Allahgholipour, F.; Atashi, H., Para-Xylene Adsorption Separation Process Using Nano-Zeolite Ba-X. *Chem. Eng. Res. Des.* **2014**, *92*, 1192-1199.

37. Rasouli, M.; Yaghobi, N.; Gilani, S. Z. M.; Atashi, H.; Rasouli, M., Influence of Monovalent Alkaline Metal Cations on Binder-Free Nano-Zeolite X in Para-Xylene Separation. *Chin. J. Chem. Eng.* **2015**, *23*, 64-70.

38. Huang, J.; Han, X.; Yang, S.; Cao, Y.; Yuan, C.; Liu, Y.; Wang, J.; Cui, Y., Microporous 3D Covalent Organic Frameworks for Liquid Chromatographic Separation of Xylene Isomers and Ethylbenzene. *J. Am. Chem. Soc.* **2019**, *141*, 8996-9003.

39. Luna, F. M. T.; Coelho, J. A.; Otoni, J. C. F.; Guimarães, A. P.; Azevedo, D. C. S.; Cavalcante, C. L., Studies of C_8 Aromatics Adsorption in Bay and Mordenite Molecular Sieves Using the Headspace Technique. *Adsorption.* **2010**, *16*, 525-530.
